# PYCR1 drives lung cancer progression through functional interactions with EGFR and TLR signaling pathways

**DOI:** 10.1038/s12276-025-01577-z

**Published:** 2025-11-18

**Authors:** Ji Hye Shin, Ji Young Kim, Mi-Jeong Kim, Yeeun Kang, Ha-Jeong Lee, Bongkum Choi, Ji Su Lee, Dohee Kwon, Seo Hyun Kim, Yoolim Sung, Duk-Hwan Kim, Jae-Hyuck Shim, Eunyoung Chun, Ki-Young Lee

**Affiliations:** 1 Department of Immunology, Samsung Biomedical Research Institute, Sungkyunkwan University School of Medicine, Suwon, Republic of Korea; 2 Division of Rheumatology, Department of Medicine, University of Massachusetts Chan Medical School, Worcester, MA USA; 3 Horae Gene Therapy Center, University of Massachusetts Chan Medical School, Worcester, MA USA; 4 Department of Medicine, Sungkyunkwan University School of Medicine, Suwon, Republic of Korea; 5 Bioanalysis Center, GenNBio Inc., Seongnam, Republic of Korea; 6 Research and Development Center, CHA Vaccine Institute, Seongnam, Republic of Korea; 7 Samsung Medical Center, Department of Health Science and Technology, Samsung Advanced Institute for Health Science and Technology, Sungkyunkwan University School of Medicine, Seoul, Republic of Korea; 8 Department of Metabiohealth, Sungkyun Convergence Institute, Sungkyunkwan University, Suwon, Republic of Korea

**Keywords:** Non-small-cell lung cancer, Non-small-cell lung cancer

## Abstract

Lung cancer, particularly non-small-cell lung cancer (NSCLC), remains a leading cause of cancer-related mortality worldwide. Recent studies have implicated pyrroline-5-carboxylate reductase 1 (PYCR1), a key enzyme in proline biosynthesis, in cancer progression, yet its specific role in lung cancer remains unclear. Here we demonstrate that PYCR1 plays a critical role in NSCLC progression through its functional association with the epidermal growth factor receptor (EGFR) and Toll-like receptor (TLR) signaling pathways. An analysis of patient datasets revealed that PYCR1 is upregulated in NSCLC tissues, with the enrichment of cancer-associated pathways in PYCR1-upregulated patients. Functional studies in *PYCR1*-knockout (*PYCR1*-KO) lung cancer cells generated via CRISPR–Cas9 showed reduced cell proliferation, migration, colony formation and tumor spheroid growth both in vitro and in vivo. Mechanistically, PYCR1 stabilizes EGFR by forming a complex with EGFR and USP11, thereby enhancing EGFR deubiquitination and stability. In addition, PYCR1 promotes TLR signaling by interacting with key downstream molecules, including TRAF6, TAK1, ECSIT and TAB2, facilitating their ubiquitination and NF-κB activation. The loss of PYCR1 attenuates EGFR- and TLR-induced signaling cascades, resulting in reduced activation of AKT, TAK1 and NF-κB. Importantly, treatment with PYCR1-IN-1, a selective PYCR1 inhibitor, significantly suppressed EGFR- and TLR-induced tumor spheroid growth in multiple lung cancer cell lines, underscoring PYCR1’s potential as a therapeutic target. Collectively, our findings establish PYCR1 as a critical regulator of EGFR and TLR signaling pathways, driving lung cancer progression. Targeting PYCR1 with pharmacological inhibitors such as PYCR1-IN-1 offers a promising strategy for combating EGFR- and TLR-driven NSCLC progression.

## PYCR1 drives non-small-cell lung cancer progression

Lung cancer is a major cause of cancer deaths, with non-small-cell lung cancer being the most common type. Researchers have found that an enzyme called pyrroline-5-carboxylate reductase 1 (PYCR1) might play a role in cancer growth, but its exact function in lung cancer is unclear. This study explores PYCR1’s role in lung cancer. The researchers used data from 42 patients with non-small-cell lung cancer to study PYCR1 levels in cancer tissues. They also created lung cancer cells without PYCR1 using CRISPR–Cas9 to see how it affects cell growth and movement. They found that removing PYCR1 reduced cancer cell growth and spread. They also tested a chemical that blocks PYCR1 and found it could slow down tumor growth. The study concludes that PYCR1 is important for lung cancer progression and could be a target for new treatments. This summary was initially drafted using artificial intelligence, then revised and fact-checked by the author.

## Introduction

Lung cancer is one of the leading causes of cancer-related deaths worldwide, with non-small-cell lung cancer (NSCLC) accounting for the majority of cases^1,2^. Despite advances in targeted therapies and immunotherapies, the prognosis for patients with advanced lung cancer remains poor, highlighting the need for novel therapeutic targets^3,4^. Recent studies have identified the enzyme pyrroline-5-carboxylate reductase 1 (PYCR1) as a potential contributor to cancer progression^5,6^, but its specific role and underlying mechanisms in lung cancer have not been fully elucidated. PYCR1 is an enzyme involved in the proline biosynthesis pathway, which is crucial for maintaining cellular redox balance and supporting rapid cell proliferation^5–8^. Importantly, elevated PYCR1 expression has been associated with various cancers^5–8^, but its role in lung cancer progression and signaling networks remains largely unexplored.

The epidermal growth factor receptor (EGFR) pathway is a well-known driver of lung cancer progression, particularly in NSCLC^9,10^. EGFR mutations and overexpression lead to enhanced cell proliferation, survival and migration through the activation of downstream signaling cascades^9–13^. Similarly, Toll-like receptors (TLRs), particularly TLR1, TLR2 and TLR4, have been implicated in cancer development by modulating the tumor microenvironment and promoting inflammatory responses^14,15^. Regarding reactive oxygen species (ROS) production, the signaling adaptor protein evolutionarily conserved signaling intermediate in Toll pathways (ECSIT), which is a critical mediator of TLR signaling, has been linked to mitochondrial function and ROS production, leading to the activation of NF-κB^16,17^. Importantly, it has been reported that mitochondrial oxidative stress mediated by Lon-PYCR1 maintains an immunosuppressive tumor microenvironment and promotes cancer progression through ROS-dependent p38 and NF-κB signaling^18^. Furthermore, TLR and EGFR signaling pathways are functionally interconnected and synergistically activate NF-κB, which is crucial for lung cancer progression^19^. Given the potential interplay between PYCR1 and these signaling pathways, we hypothesized that PYCR1 might be functionally associated with EGFR and TLR signaling in lung cancer.

To test this hypothesis, we analyzed PYCR1 expression levels in lung cancer tissues using the Gene Expression Profiling Interactive Analysis (GEPIA) database and confirmed our findings with cohort microarray datasets from 42 patients with NSCLC. Based on pathway enrichment analysis in PYCR1-upregulated patients with NSCLC, we performed biochemical study and identified the functional role of PYCR1 in EGFR- and TLR-mediated signaling. To verify the functional role of PYCR1 in lung cancer, we generated *PYCR1*-knockout (*PYCR1*-KO) human lung cancer cells using CRISPR–CRISPR-associated protein 9 (Cas9) gene editing to assess the impact of PYCR1 loss on cell proliferation, migration and tumor formation in both in vitro and in vivo. Furthermore, we evaluated the efficacy of PYCR1-IN-1, a chemical inhibitor of PYCR1, in EGFR- or TLR-driven NSCLC models. Collectively, our results demonstrate that PYCR1 is a key regulator of lung cancer progression and is functionally linked to both EGFR and TLR signaling pathways. These findings provide a foundation for future studies aimed at developing PYCR1-targeted therapies for clinical application in patients with NSCLC.

## Materials and methods

### Patients and samples

This study was conducted in accordance with the ethical principles stated in the Declaration of Helsinki. It was approved by the Institutional Review Board (IRB no. 2010-07-204) of Samsung Medical Center (SMC, Seoul, Korea). Written informed consent to use pathological specimens for research was obtained from all patients before surgery. Lung tumor tissues and matched lung normal tissues of patients with NSCLC (*n* = 42) who had been diagnosed with lung cancer were obtained from SMC. Lung tumor and matched normal specimens of enrolled patients were immediately frozen in liquid nitrogen and stored at −80 °C until use.

### Xenografted NSG mouse model

NOD/SCID/IL-2Rγnull (NSG) mice were purchased from the Jackson Laboratory and maintained under specific pathogen-free conditions in accordance with ethical guidelines for the care of these mice at the Bioanalysis Center Animal Facility, GenNBio. All experimental procedures were approved by the Institutional Animal Care and Use Committee (IACUC) of the Bioanalysis Center Animal Facility (IACUC no. 23-10-01). NSG mice at 6–8 weeks old were used to generate xenografted NSG mice. Control (Ctrl) A549 (5 × 10^6^ cells per mouse, *n* = 4) or *PYCR1*-KO A549 cells (5 × 10^6 ^cells per mouse, *n* = 4) in serum-free Roswell Park Memorial Institute 1640 medium were injected under NSG mice skin (back area)^20,21^. The final injection volume was 100 μl per mouse containing a 1:1 v/v mixture of ice-chilled Matrigel (BD Biosciences), which was kept on ice until injection. After injecting cancer cells, tumor volume was measured with a caliper until 53 days after injection. Tumor volumes (in mm^3^) were calculated as length × (width)^2^ × 0.5. Tumor growth curves are presented as average tumor volume ± s.e.m. for each group in this study. All studies involving mice were approved by the Nemours IACUC.

### Cells

A549 cells (human lung cancer cell line; CCL-185, American Type Culture Collection (ATCC)), H1299 cells (human NSCLC cell line; CRL-5803, ATCC), H460 cells (human large-cell lung cancer cell line; HTB-177), H358 cells (human bronchioalveolar carcinoma cell line; CRL-5807), H1975 cells (human lung cancer cell line harboring the L858R/T790M EGFR mutations; CRL-5908, ATCC) and HCC827 cells (human NSCLC cell line carrying an EGFR exon 19 deletion; KCLB no. 60508, Korean Cell Line Bank (KCLB)) were maintained in Roswell Park Memorial Institute 1640 medium (LM011-01, Welgene) supplemented with 10% fetal bovine serum (FBS), penicillin (100 μg/ml) and streptomycin (100 μg/ml) in a 5% CO_2_ humidified atmosphere at 37 °C. Human embryonic kidney (HEK) 293T cells (CRL-11268, ATCC) were cultured and maintained in Dulbecco’s modified Eagle medium (LM001-05, Welgene) with 10% FBS.

### Antibodies and reagents

Anti-PYCR1 (13108-1-AP) antibody was purchased from Proteintech. PYCR1-IN-1 (HY-126271), and mitoxantrone (HY-13502) were purchased from MedChemExpress. The anti-Flag (F3165) antibody was purchased from Sigma-Aldrich. Anti-TRAF6 (8028S), anti-EGFR (2232S), anti-pho-EGFR (2236S), anti-pho-TAK1 (Thr184/187) (4508S), anti-pho-IKKα/β (Ser176/180) (2697S), anti-p-NF-κB p65 (S536) (3033S), anti-IKKβ (2684S) and anti-Akt1 (75692S) antibodies were purchased from Cell Signaling Technology. Anti-GAPDH (sc-47724), antip-pho-Akt1 (sc-52940), anti-Myc (sc-40), anti-IRAK1 (sc-5288), anti-Ubiquitin (sc-8017), anti-TAK1 (sc-7967) and anti-HA tag (sc-7392) antibodies were purchased from Santa Cruz Biotechnology. Anti-NF-κB p65/RelA (A2547) and anti-TAB2 (A9867) antibodies were purchased from ABclonal. Anti-ECSIT (ab21288) and Rabbit anti-mouse IgG H&L (HRP) (ab6728) antibodies were purchased from Abcam. Goat anti-rabbit IgG (HRP) (GTX213110-01) antibody was purchased from GeneTex. TrueBlot secondary antibodies (18-8816-33, 18-8817-33) were purchased from Rockland Immunochemicals. Heat-killed *Listeria monocytogenes* (HKLM, tlrl-hklm), Pam3CysSerLys4 (Pam3csk4, tlrl-pms) and polyinosinic:polycytidylic acid (Poly(I:C), tlrl-pic) were purchased from InvivoGen. Lipopolysaccharide (LPS; L3024), dimethyl sulfoxide (DMSO; D4540), Dulbecco’s phosphate-buffered saline (D8537), glutaraldehyde (G6257-100ml), crystal violet (C6158-50g), EGF (SRP3027) and Thiazolyl blue tetrazolium bromide (MTT; M5655) were purchased from Sigma-Aldrich. Lipofectamine 2000 (11668019) and Opti-MEM (31985070) were purchased from Thermo Fisher Scientific. Agarose powder (AGA001.500) was purchased from Bioshop Canada. Protein G beads (17-0618-02) was purchased from GE HealthCare. The Transwell with 8.0-µm pore polycarbonate membrane insert (3422) was purchased from Corning.

### Plasmid constructs

EGFR wild-type (WT) (11011), W118-1 Flag-hIRAK1 (180405), Flag-TRAF6 (21624), pRK6-HA-TAK1 (44160) and Flag-HA-ubiquitin-specific protease 11 (USP11) (22566) were purchased from Addgene. Flag-ECSIT and Flag-TAB2 vectors were generated as previously described^22,23^. pCMV-3Tag-7 (240202) and pCMV-3Tag-6 (240200) were purchased from Agilent Technologies. The full-length of PYCR1 was generated by PCR using a complementary DNA (cDNA) library as a template and inserted into the pCMV-3Tag 6 or pCMV-3Tag-7 vector to generate the Flag-PYCR1 or Myc-PYCR1 vector, respectively. The full-length of ECSIT, TAB2, EGFR, TAK1, TRAF6, IRAK1 and USP11 was cloned into pCMV-3Tag-7 or pCMV-3Tag-6 vectors to generate various Flag- or Myc-tagged constructs. The truncated mutants, including Flag-EGFR (669–1210 and 954–1210), Myc-TAK1 (1–300, 1–400 and 1–500), Flag-TRAF6 (110–522, 260–522 and 349–522) and Myc-ECSIT (1–200 and 1–300), were generated by PCR using the respective WT vector as a template and inserted into pCMV-3Tag-7 or pCMV-3Tag-6 vectors.

### Generation of *PYCR1*-KO cell lines with CRISPR–Cas9

To generate *PYCR1*-KO lung cancer cells with the CRISPR–Cas9 gene editing method, we used two vector systems, including single guide RNA (sgRNA) and Cas9 vectors^24–29^. The sgRNA and Cas9 vectors were kindly provided by Dr. Daesik Kim (Sungkyunkwan University School of Medicine, Suwon, Korea). The guide RNA (gRNA) sequences for CRISPR–Cas9 were designed on the CRISPR design website (http://crispr.mit.edu/) provided by the Feng Zhang Lab. The insert oligonucleotides for human PYCR1 gRNA were: 5′-TGAAATAGGCGCCGACATTG-3′ (gRNA-1)/5′-CTTGGCTGCCCACAAGATAA-3′ (gRNA-2)/5′-GCTGCACCGTCTCCTTGTTG-3′ (gRNA-3)/5′-GGGAGCTAGCCATTATCTTG-3′ (gRNA-4). The complementary oligonucleotides to gRNAs were annealed and cloned into a sgRNA vector. The sgRNA vectors expressing gRNA of PYCR1 and Cas9 vector expressing Cas9 were transfected into A549 cells and H1299 cells using Lipofectamine 2000 (Thermo Fisher Scientific) according to the manufacturer’s instructions. After 2 weeks, the colonies were isolated from 96-well plates and expression levels of PYCR1 was analyzed with western blotting.

### IP assay

The HEK-293T cells were transiently transfected with mock (a relevant control vector), Flag-PYCR1, Myc-PYCR1, Flag-USP11, Myc-USP11, Flag-TRAF6, Flag-TRAF6 truncated mutants, Flag-ECSIT, Myc-ECSIT, Myc-ECSIT truncated mutants, Flag-TAB2, Flag-TAK1, Myc-TAK1, Myc-TAK1 truncated mutants, Flag-EGFR, Flag-EGFR truncated mutants or Flag-IRAK1 as indicated in each figure for 24 h. After collecting the cells, the cell lysates were prepared and immunoprecipitated with anti-Flag or anti-Myc antibody. The immunoprecipitation (IP) complexes were separated by sodium dodecyl sulfate–polyacrylamide gel electrophoresis (SDS–PAGE, 8–12%) and immune-probed with anti-Myc or anti-Flag antibodies. For semiendogenous IP, the A549 WT cells were transiently transfected with mock or Flag-PYCR1 for 24 h and then stimulated with vehicle or LPS (20 μg/ml) for 6 h. H1299 WT cells were transiently transfected with Flag-PYCR1 for 24 h. After collecting the cells, the cell lysates were prepared and immunoprecipitated with anti-IgG or anti-Flag antibody. The IP complexes were separated by SDS–PAGE (8–12%) and immune-probed with anti-TRAF6, anti-TAK1, anti-TAB2, anti-ECSIT, anti-IRAK1, anti-EGFR or anti-Flag antibodies.

### Ubiquitination assay

The HEK-293T cells were transiently transfected with mock (a relevant control vector), HA-Ub, Flag-PYCR1, Myc-PYCR1, Flag-EGFR and Myc-USP11 as indicated in each figure for 24 h. After collecting the cells, the cell lysates were prepared and immunoprecipitated with anti-Flag antibody. The IP complexes were separated by SDS–PAGE (8–12%) and immune-probed with anti-Myc or anti-Flag antibodies. For semiendogenous IP, A549 WT or H1299 WT cells were transiently transfected with mock (a relevant control vector), Flag-TRAF6, Flag-ECSIT, Flag-TAK1 or Myc-PYCR1 for 24 h. After collecting cells, cell lysates were prepared and immunoprecipitated with anti-Flag antibody. IP complexes were separated by SDS–PAGE (8–12%) and immune-probed with anti-Ubiquitin, anti-Myc or anti-Flag antibodies.

### Western blotting assay

WT A549 or H1299 lung cancer cells were seeded into 12-well plates and cultured. Cells were stimulated with vehicle (DMSO, 0.1% v/v concentration) or different concentrations of mitoxantrone (USP11 inhibitor) for 24 h. After collecting the cells, the cell lysates were separated by SDS–PAGE (~8–12%) and immune-probed with anti-EGFR or anti-GAPDH (as loading control) antibodies. The WT lung cancer cells were seeded into 12-well plates and cultured. The cells were transfected with mock (a relevant control vector) or Flag-PYCR1. After 24 h of incubation, the transfected cells were stimulated with vehicle (DMSO, 0.1% v/v concentration) or LPS (15 μg/ml) for 6 h. After collecting the cells, the cell lysates were separated by SDS–PAGE (~8–12%) and immune-probed with anti-Flag, anti-pho-p65, anti-p65, anti-pho-IKKs, anti-IKKs, anti-pho-TAK1 (Thr184/187), anti-TAK1 or anti-GAPDH (as loading control) antibodies. Ctrl A549 and *PYCR1*-KO A549 lung cancer cells were seeded into 12-well plates and cultured. The cells were treated with vehicle (DMSO, 0.1% v/v concentration), Pam3CSK4 (3 μg/ml), LPS (10 μg/ml) or EGF (10 ng/ml) for different time periods. After collecting the cells, the cell lysates were separated by SDS–PAGE (~8–12%) and immune-probed with anti-pho-p65, anti-p65, anti-pho-IKKs, anti-IKKs, anti-pho-TAK1 (Thr184/187), anti-TAK1, anti-pho-EGFR (Y1068), anti-EGFR, anti-pho-AKT1, anti-AKT1 or anti-GAPDH (as loading control) antibodies.

### Transwell migration assay

The Transwell migration assay was performed as previously described^24–30^. In brief, Ctrl A549, *PYCR1*-KO A549, Ctrl H1299 and *PYCR1*-KO H1299 lung cancer cells were suspended in a culture medium (250 μl) and added to the upper compartment of a 24-well Transwell chamber (8-μm pore; Corning, 3422). Ctrl A549, *PYCR1*-KO A549, Ctrl H1299 and *PYCR1*-KO H1299 lung cancer cells and free culture medium (250 μl) were mixed with vehicle (DMSO, 0.1% v/v concentration), Pam3CSK4 (3 μg/ml), LPS (3 μg/ml) or EGF (15 ng/ml) and incubated at 37 °C for 24 h. The migratory cells would pass through polycarbonate membranes and cling to the bottom side. The nonmigratory cells would stay in the upper chamber. After removing the nonmigratory cells, the migratory cells were fixed using 2.5% glutaraldehyde (Sigma-Aldrich, G6257-100 ml) and then stained with 0.1% crystal violet (Sigma-Aldrich, C6158-50g).

### Wound-healing migration assay

The wound-healing migration assay was performed as following protocols^24–30^. In brief, Ctrl A549, *PYCR1*-KO A549, Ctrl H1299 and *PYCR1-*KO H1299 cells were seeded into 12-well plates and cultured to reach confluence. The cell monolayers were gently scratched and washed with a culture medium. After the floating cells and debris were removed, the cells attached to culture plates were treated with vehicle (DMSO, 0.1% v/v concentration), Pam3CSK4 (3 μg/ml), LPS (10 μg/ml) or EGF (15 ng/ml) for different time periods. The cell images were captured after culturing for different periods as indicated in each experiment.

### Anchorage-independent soft agar colony formation assay

The anchorage-independent soft agar colony formation assay was performed as following protocols^24–30^. In brief, Ctrl A549, *PYCR1*-KO A549, Ctrl H1299 and *PYCR1-*KO H1299 cells mixed with 0.3% agarose (Biotechnology Grade, GA001.500) in complete medium were plated onto the bottom of a 0.5% agar layer in a six-well plate with a complete medium. Growth medium (2 ml) with vehicle (DMSO, 0.1% v/v concentration), Pam3CSK4 (1 μg/ml), LPS (10 μg/ml) or EGF (20 ng/ml) was added on top of the layer and cells were incubated at 37 °C for 42 days.

### Anchorage-dependent colony formation assay

The ability of a single cell to grow into a colony was determined as following protocols^24–30^. In brief, Ctrl A549, *PYCR1*-KO A549, Ctrl H1299 and *PYCR1*-KO H1299 cells were collected with trypsin–ethylenediaminetetraacetic acid and resuspended in a singular form. Then, 700 cells per well were plated into a six-well plate and treated with vehicle (DMSO, 0.1% v/v concentration), Pam3CSK4 (3 μg/ml), LPS (5 μg/ml) or EGF (20 ng/ml). After incubation for 8 days, the colonies were stained with 0.5% crystal violet (Sigma-Aldrich, C6158-50g) for 30 min at room temperature.

### MTT assay

The Ctrl A549, *PYCR1*-KO A549, Ctrl H1299 and *PYCR1*-KO H1299 cells were seeded into 96-well culture plates at a density of 700 cells per well, treated with vehicle (DMSO, 0.1% v/v concentration), Pam3CSK4 (1 μg/ml), LPS (2.5 μg/ml) or EGF (10 ng/ml) and grown in a culture medium supplemented with 10% FBS for different time periods. Cell viability was measured using an MTT reagent (Sigma-Aldrich, M5655) dissolved in phosphate-buffered saline (1 mg/ml). On the measurement day, the medium was carefully replaced with Dulbecco’s phosphate-buffered-saline-diluted MTT (1:10, 10% MTT) and incubated for 3 h at 37 °C. After incubation, the medium was removed, and the formazan crystals were dissolved in 100 µl of DMSO. The MTT reduction was quantified by measuring absorbance at 595 nm using a Bio-Rad Model 680 microplate reader (Bio-Rad). Each test was repeated at least four times in quadruplicate.

### NF-κB luciferase reporter assay

A549 cells were transiently transfected with mock or different concentrations of Flag-PYCR1 together with the pBIIx-luc NF-κB-dependent reporter construct and the *Renilla luciferase* vector. At 36 h post transfection, the cells were either treated with LPS (100 ng/ml) or left untreated for 6 h and lysed, and luciferase activity was measured using a dual-luciferase assay kit (Promega).

### 3D spheroids formation assay using agarose-coated plates

The three-dimensional (3D) spheroid formation assay was performed following protocol^20,25^. In brief, 1.5% agarose hydrogel was added to each well of a 96-well culture plate and incubated at room temperature for 30 min. Ctrl A549, *PYCR1*-KO A549, Ctrl H1299 and *PYCR1*-KO H1299 cells were seeded into 100 µl of growth medium at a density of 125 or 500 cells per well. The plates were incubated at 37 °C for 48 h to allow spheroid formation. The spheroids were then treated with vehicle (DMSO, 0.1% v/v), Pam3CSK4 (2 µg/ml), LPS (5 µg/ml) or EGF (10 ng/ml) and incubated for additional time periods. WT A549 or H1299 lung cancer cells were seeded into 12-well plates and transfected with mock (control vector) or Flag-PYCR1. After 24 h of incubation, transfected cells were seeded into 100 µl of growth medium at a density of 125 or 500 cells per well. The plates were incubated at 37 °C for 48 h to allow spheroid formation. The spheroids were then treated with vehicle (DMSO, 0.1% v/v), Pam3CSK4 (2 µg/ml), LPS (5 µg/ml) or EGF (10 ng/ml) and incubated for additional time periods. Spheroid formation and growth were assessed using phase-contrast microscopy, and spheroid size was measured using ImageJ Software (National Institutes of Health). To evaluate the inhibitory effect of PYCR1-IN-1 on spheroid formation in A549, H1299 and H460 cells, these cells were seeded into 96-well plates at a density of 50 or 250 cells per well and incubated at 37 °C for 48 h to allow spheroid formation. The spheroids were then treated with vehicle (DMSO, 0.1% v/v) or 10 µM PYCR1-IN-1. After 24 h, spheroids were further treated with Pam3CSK4 (3 µg/ml), HKLM (10⁷/ml), Poly(I:C) (5 µg/ml), LPS (5 µg/ml) or EGF (10 ng/ml). The tumor spheroid formation and growth were evaluated using phase-contrast microscopy, and spheroid sizes were measured using ImageJ Software.

### Microarray analysis

The microarray analysis was performed as previously described^31–33^. From tumor and matched normal tissues of 42 patients with NSCLC, total RNAs were extracted with Trizol (Thermo Fisher Scientific, 15596026) and purified using RNeasy columns (Qiagen, 74106) according to each manufacturer’s protocol.

### GSEA

**Fig. 1:**
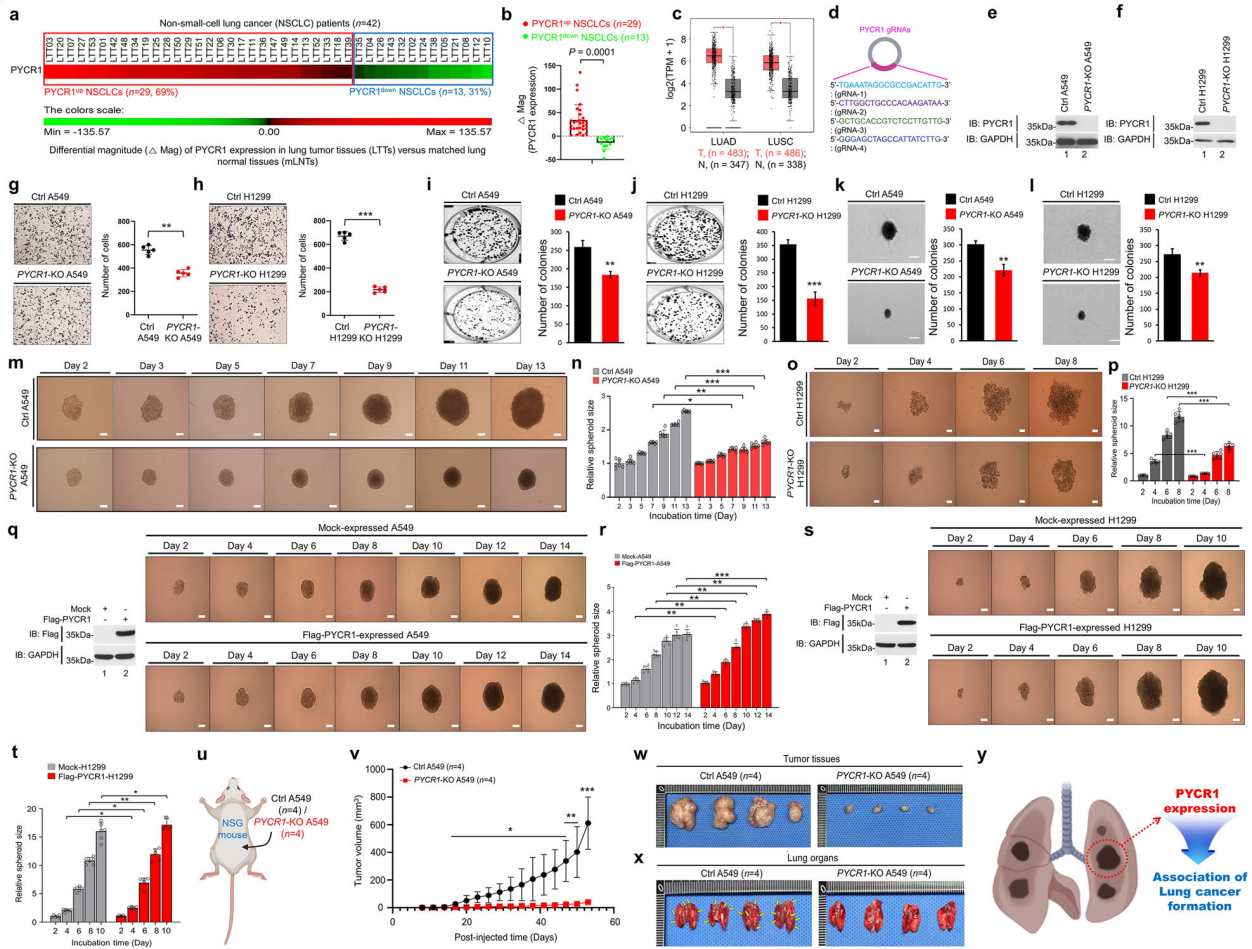
PYCR1 expression is associated in patients with NSCLC and involved in tumorigenesis. **a**, **b** The ΔMag of PYCR1 expression was represented in 42 patients with NSCLC (**a**); the scatter plots show the relative levels of ΔMag PYCR1 in PYCR1^up^ patients with NSCLC (*n* = 29) compared with PYCR1^down^ patients with NSCLC (*n* = 13) (**b**). Based on the ΔMag of PYCR1 expression, 42 patients with NSCLC were categorized into two groups: PYCR1^up^ (*n* = 29, red box) and PYCR1^down^ (*n* = 13, blue box). **c** The scatter plots illustrate the relative levels of PYCR1 mRNA in nontumor (N) and tumor tissues (T). One-way ANOVA; *P < 0.01. **d** Four different gRNAs were designed to create *PYCR1*-KO lung cancer cells using the CRISPR–Cas9 gene editing method. **e**, **f** The *PYCR1*-KO A549 (**e**) and *PYCR1*-KO H1299 (**f**) lung cancer cells were generated. The endogenous expression of PYCR1 was verified by western blotting using anti-PYCR1 and anti-GAPDH antibodies. **g**, **h** The Transwell migration assay was performed on Ctrl A549 and *PYCR1*-KO A549 cells (**g**) or Ctrl H1299 and *PYCR1*-KO H1299 cells (**h**). The absolute number of migrated cells is presented as the mean ± s.d. (*n* = 5). **i**, **j** The anchorage-dependent colony formation assay was conducted on Ctrl A549 and *PYCR1*-KO A549 cells (**i**) or Ctrl H1299 and *PYCR1*-KO H1299 cells (**j**). The number of colonies is presented as the mean ± s.d. (*n* = 5). **k**, **l** The anchorage-independent soft agar colony formation assay was performed on Ctrl A549 and *PYCR1*-KO A549 cells (**k**) or Ctrl H1299 and *PYCR1*-KO H1299 cells (**l**). The number of colonies is presented as the mean ± s.d. (*n* = 5). **m**–**p** The 3D spheroid formation assay was performed on Ctrl A549 and *PYCR1*-KO A549 cells (500 cells per well) (**m**, phase-contrast microscopy images) or Ctrl H1299 and *PYCR1*-KO H1299 cells (125 cells per well) (**o**, phase-contrast microscopy images). The plates were incubated at 37 °C for 48 h to allow the 3D spheroid formation. The spheroids were incubated for various periods, as indicated. The representative images of spheroids were captured using phase-contrast microscopy (scale bar, 100 μm). The size of the spheroids was assessed using ImageJ Software. The error bars represent ± s.d. (*n* = 7) (**n**, spheroid sizes in Ctrl A549 and *PYCR1*-KO A549; **p**, spheroid sizes in Ctrl H1299 and *PYCR1*-KO H1299). **q**–**t** The WT A549 or WT H1299 cells were transfected with the mock control vector or Flag-PYCR1; the Flag-PYCR1 expression was confirmed by western blotting using anti-Flag or anti-GAPDH as a loading control (A549 cells (left) (**q**) and H1299 cells (left) (**s**)). The 3D tumor spheroid formation assay was performed on mock-expressed A549 and Flag-PYCR1-expressed A549 cells (in **q**, phase-contrast microscopy images; scale bar, 100 μm) or mock-expressed H1299 and Flag-PYCR1-expressed H1299 cells (**s**, phase-contrast microscopy images; scale bar, 100 μm) for different time periods, as indicated in each figure. The size of the spheroids was assessed using ImageJ Software. The error bars represent ± s.d. (**r** and **t**, spheroid sizes; *n* = 6). *P < 0.05, **P < 0.01, ***P < 0.001. **u**–**x** Ctrl A549 (5 × 10^6 ^cells per mouse, *n* = 4) or *PYCR1*-KO A549 cells (5 × 10^6 ^cells per mouse, *n* = 4) were injected under the skin of NSG mice (back area) (**u**); tumor growth curves are presented as average tumor volume ± s.e.m. (**v**), and the tumor tissues (**w**) and lung organs (**x**, metastatic nodules indicated as arrows). The tumor volumes were measured with a caliper until 53 days post injection. *P < 0.05, **P < 0.01, ***P < 0.001. **y** A schematic illustration showing the role of PYCR1 expression in promoting tumorigenesis and its association with lung cancer formation.

Different magnitudes of PYCR1, EGFR, TRAF6, TAK1, TAB2, TLR4, TLR2 and TLR1 expression were obtained from normalized microarray data between lung tumor tissues and matched lung normal tissues. Based on the expression of PYCR1, EGFR and PYCR1; TRAF6, TAK1, TAB2 and PYCR1; or TLR4, TLR2, TLR1 and PYCR1, 42 patients with NSCLC were stratified into upregulated patients and downregulated patients, as indicated in Fig. 1a, Supplementary Fig. 4a, Fig. 4g, and Fig. 5a. The genes showing substantial differences, such as normalized enrichment score (NES) and nominal *P* value were analyzed by gene set enrichment analysis (GSEA) (http://www.gsea-msigdb.org/gsea/index.jsp).

### The Cancer Genome Atlas data analysis

The expression of PYCR1 in tumor and normal tissues was analyzed using The Cancer Genome Atlas (TCGA) data (GEPIA; http://gepia.cancer-pku.cn/detail.php?gene=PYCR1).

### Statistical analysis

All data are expressed as mean ± s.d. Statistical significance was determined by Student’s *t*-test using GraphPad Prism 5.0 (GraphPad Software). *P* values are marked as *P < 0.05, **P < 0.01, ***P < 0.001, ****P < 0.0001, ^#^*P* < 0.05, ^##^*P* < 0.01, ^###^*P* < 0.001 and ^####^*P* < 0.0001.

## Results

### PYCR1 drives lung cancer progression by promoting cell proliferation, migration, tumor spheroid formation, metastasis and tumorigenicity

To investigate the relationship between PYCR1 expression and NSCLC, we analyzed a microarray dataset comprising lung tumor tissues (*n* = 42) and their matched lung normal tissues (*n* = 42) (Supplementary Fig. 1a). Based on the differential magnitude (ΔMag) of the average normalized PYCR1 signal values between the lung tumor tissues and matched lung normal tissues, patients with NSCLC were categorized into two groups: PYCR1-upregulated (PYCR1^up^) and PYCR1-downregulated (PYCR1^down^) (Fig. 1a and Supplementary Table 1). Among the 42 patients with NSCLC, PYCR1 expression was upregulated in 29 patients (69%) and downregulated in 13 patients (31%) (Fig. 1a), with a significant ΔMag difference between the PYCR1^up^ and PYCR1^down^ groups (Fig. 1b). These results were further validated using public data from the GEPIA platform, which includes datasets for lung adenocarcinoma (LUAD) and lung squamous cell carcinoma (LUSC) (Fig. 1c and Supplementary Fig. 1b). To determine whether cancer-related and oncogenic pathways were differentially enriched between patients with PYCR1^up^ NSCLC (*n* = 29) and patients with PYCR1^down^ NSCLC (*n* = 13), we performed GSEA. The analysis revealed a notable enrichment of cancer-related gene modules, including pathways associated with matrix metalloproteinases (MMPs), phosphatase regulators and small monomeric GTPases, which play essential roles in cancer development and progression^34–36^ (Supplementary Fig. 2a–m). Furthermore, gene sets related to NSCLC and broader cancer-related pathways were remarkably enriched in patients with PYCR1^up^ NSCLC (Supplementary Fig. 2n, o), providing strong evidence for the association between elevated PYCR1 expression and lung cancer progression.

To validate the functional role of PYCR1 in lung cancer, we generated *PYCR1*-KO human lung cancer cell lines using CRISPR–Cas9 gene editing (Fig. 1d–f). The KO of PYCR1 significantly reduced Transwell migration in both A549 and H1299 cells compared with control cells (Fig. 1g, h). The colony formation assays demonstrated that both anchorage-dependent and anchorage-independent colony formation were significantly decreased in *PYCR1*-KO lung cancer cells (Fig. 1i, j, anchorage-dependent; Fig. 1k, l, anchorage-independent). Consistently, cell proliferation was markedly reduced in *PYCR1*-KO A549 and *PYCR1*-KO H1299 cells (Supplementary Fig. 3a, b), indicating that PYCR1 supports the proliferation and migration of lung cancer cells. Further investigation using a 3D tumor spheroid formation assay revealed significantly smaller tumor spheroids in *PYCR1*-KO A549 and *PYCR1*-KO H1299 cells compared with control cells (Fig. 1m–p). Conversely, the overexpression of Flag-PYCR1 in A549 and H1299 cells resulted in significantly larger spheroids compared with mock-transfected control cells (Fig. 1q–t). The in vivo experiments confirmed these findings. The tumor size was significantly reduced in NSG mice xenografted with *PYCR1*-KO A549 cells compared with those xenografted with Ctrl A549 cells (Fig. 1u–w). Moreover, the metastasis to lung tissues was significantly diminished in NSG mice xenografted with *PYCR1*-KO A549 cells (Fig. 1x). These results demonstrate that, as illustrated in Fig. 1y, PYCR1 plays a critical role in promoting lung cancer formation by enhancing cell proliferation, migration, tumor spheroid formation, metastasis and tumorigenicity.

### PYCR1 enhances EGFR stability through interaction with USP11

**Fig. 2:**
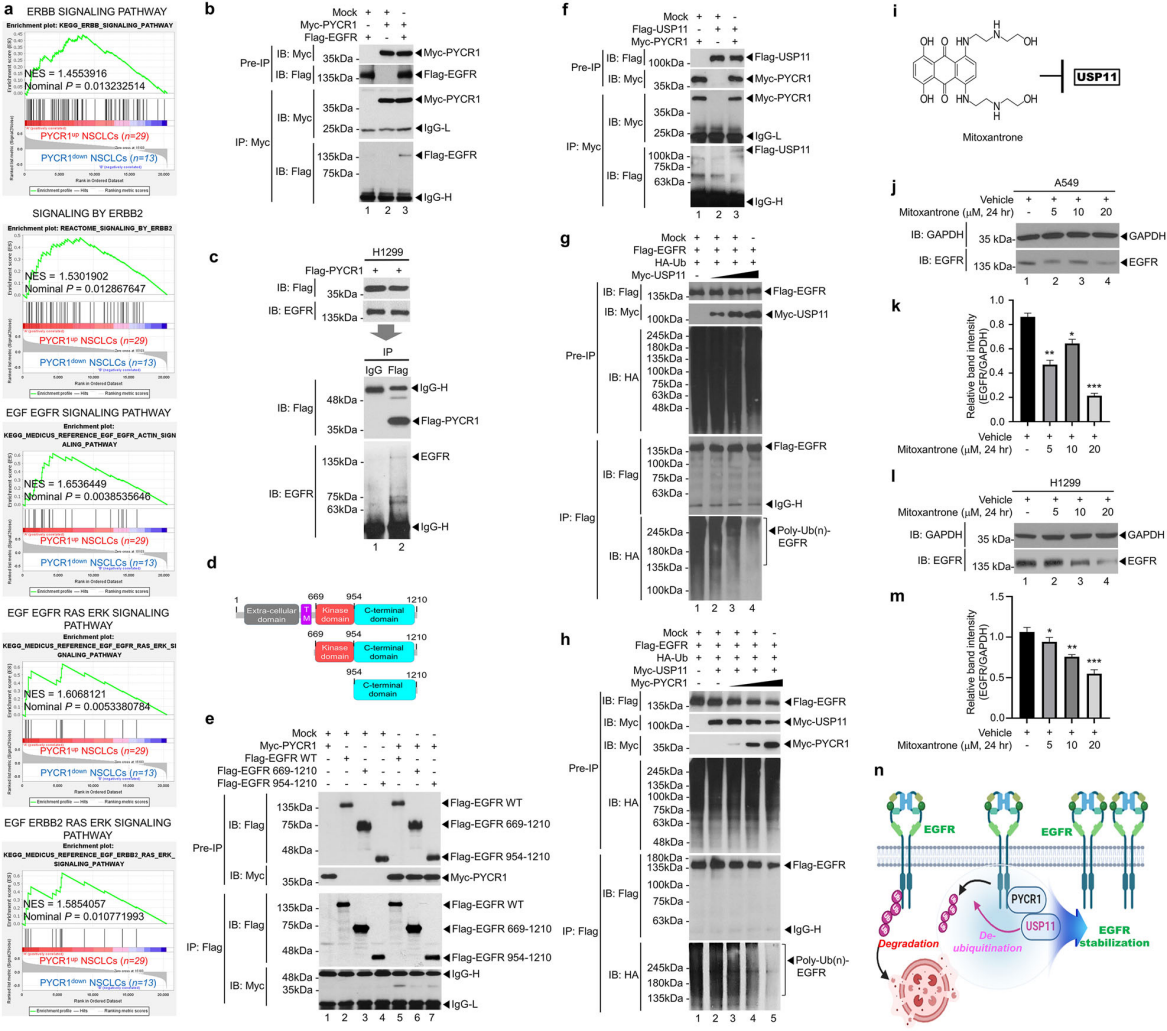
PYCR1 stabilizes EGFR by preventing its ubiquitination, thereby promoting EGFR signaling pathways. **a** A GSEA was conducted between the 29 patients with PYCR1^up^ NSCLCs (red) and the 13 patients with PYCR1^down^ NSCLCs (blue). The gene sets related to the EGFR signaling pathway were represented. The NES and the nominal *P* value are indicated in the inner section. **b** The HEK-293T cells were transiently transfected with mock (control vector), Myc-PYCR1 or Flag-EGFR, as indicated. An IP assay was performed with an anti-Myc antibody, followed by an immunoblotting (IB) assay with anti-Flag or anti-Myc antibodies. **c** The H1299 lung cancer cells were transiently transfected with Flag-PYCR1. An IP assay was performed with anti-IgG or anti-Flag antibodies, followed by an IB assay with anti-Flag or anti-EGFR antibodies. **d** The truncated mutants of EGFR were generated and presented. **e** The HEK-293T cells were transiently transfected with mock, Myc-PYCR1 or Flag-EGFR WT and its truncated mutants, as indicated. An IP assay was performed with an anti-Flag antibody, followed by an IB assay with anti-Flag or anti-Myc antibodies. **f** The HEK-293T cells were transiently transfected with mock, Myc-PYCR1 or Flag-USP11, as indicated. The IP assay was performed with an anti-Myc antibody, followed by an IB assay with anti-Flag or anti-Myc antibodies. **g** The HEK-293T cells were transiently transfected with mock, HA-Ub, Myc-USP11 or Flag-EGFR, as indicated. The IP assay was performed with anti-Flag antibodies, followed by an IB assay with anti-Flag, anti-Myc or anti-HA antibodies. **h** The HEK-293T cells were transiently transfected with mock, Flag-EGFR, HA-Ub, Myc-USP11 or different concentrations of Myc-PYCR1, as indicated. IP assay was performed with anti-Flag antibodies, followed by an IB assay with anti-Flag, anti-Myc or anti-HA antibodies. **i** The chemical structure of mitoxantrone is represented to be used as a USP11 inhibitor. **j**–**m** The USP11 inhibitor mitoxantrone was treated to A549 (**j**, western blotting for EGFR; **k**, EGFR band intensity) and H1299 (**l**, western blotting for EGFR; **m**, EGFR band intensity) cells. The EGFR protein levels were assessed by western blotting using an anti-EGFR antibody. The error bars represent ± s.d. (*n* = 3). *P < 0.05, **P < 0.01, ***P < 0.001. **n** A schematic model illustrates that PYCR1 interacts with USP11, which facilitates the deubiquitination of EGFR, leading to its stabilization.

Notably, patients with NSCLC with upregulated PYCR1 expression exhibited the substantial enrichment of gene sets related to EGFR-associated pathways compared with patients with downregulated PYCR1 expression (Fig. 2a), supposing a functional association between PYCR1 and EGFR signaling in NSCLC. To investigate the functional linkage between PYCR1 and EGFR, biochemical analyses were conducted. PYCR1 was found to interact with EGFR (Fig. 2b), and it was confirmed in H1299 cells through a semiendogenous IP assay (Fig. 2c). To determine the specific domain of EGFR involved in its interaction with PYCR1, truncated EGFR mutants were generated (Fig. 2d). The IP assays revealed that PYCR1 interacted with the WT EGFR as well as the truncated mutants EGFR 669–1210 and EGFR 954–1210 (Fig. 2e), indicating that the interaction occurs via the C-terminal domain of EGFR.

A recent report has shown that USP11 promotes partial epithelial–mesenchymal transition (EMT) by deubiquitinating the EGFR during kidney fibrosis^37^. Moreover, USP11 enhances ROS production in endothelial cells following ionizing radiation^38^. This led us to hypothesize that PYCR1 may facilitate USP11-mediated EGFR stabilization. Supporting this idea, PYCR1 was found to interact with USP11 (Fig. 2f) and enhance EGFR deubiquitination in a dose-dependent manner (Fig. 2g). Furthermore, PYCR1 remarkably enhanced EGFR deubiquitination in the presence of USP11 in a dose-dependent manner (Fig. 2h). To directly examine whether USP11 regulates EGFR expression, we used mitoxantrone, a USP11 inhibitor^39^ (Fig. 2i). The mitoxantrone treatment significantly reduced EGFR levels in both A549 and H1299 lung cancer cells, with a dose-dependent effect (Fig. 2j–m). Moreover, the association study between PYCR1 and EGFR expression in patients with NSCLC remarkably showed that gene sets related to cancer modules, cancer progression and EGFR signals were highly enriched in 20 patients with PYCR1^up^EGFR^up^ NSCLC as compared with those of 3 patients with PYCR1^down^EGFR^down^ NSCLC (Supplementary Table 2 and Supplementary Fig. 4a–q). These results strongly indicate that PYCR1 interacts with both EGFR and USP11, enhancing EGFR stability by promoting USP11-mediated EGFR deubiquitination, as illustrated in Fig. 2n.

### PYCR1 facilitates TLR-mediated signaling by promoting TRAF6, TAK1 and ECSIT ubiquitination for NF-κB activation

**Fig. 3:**
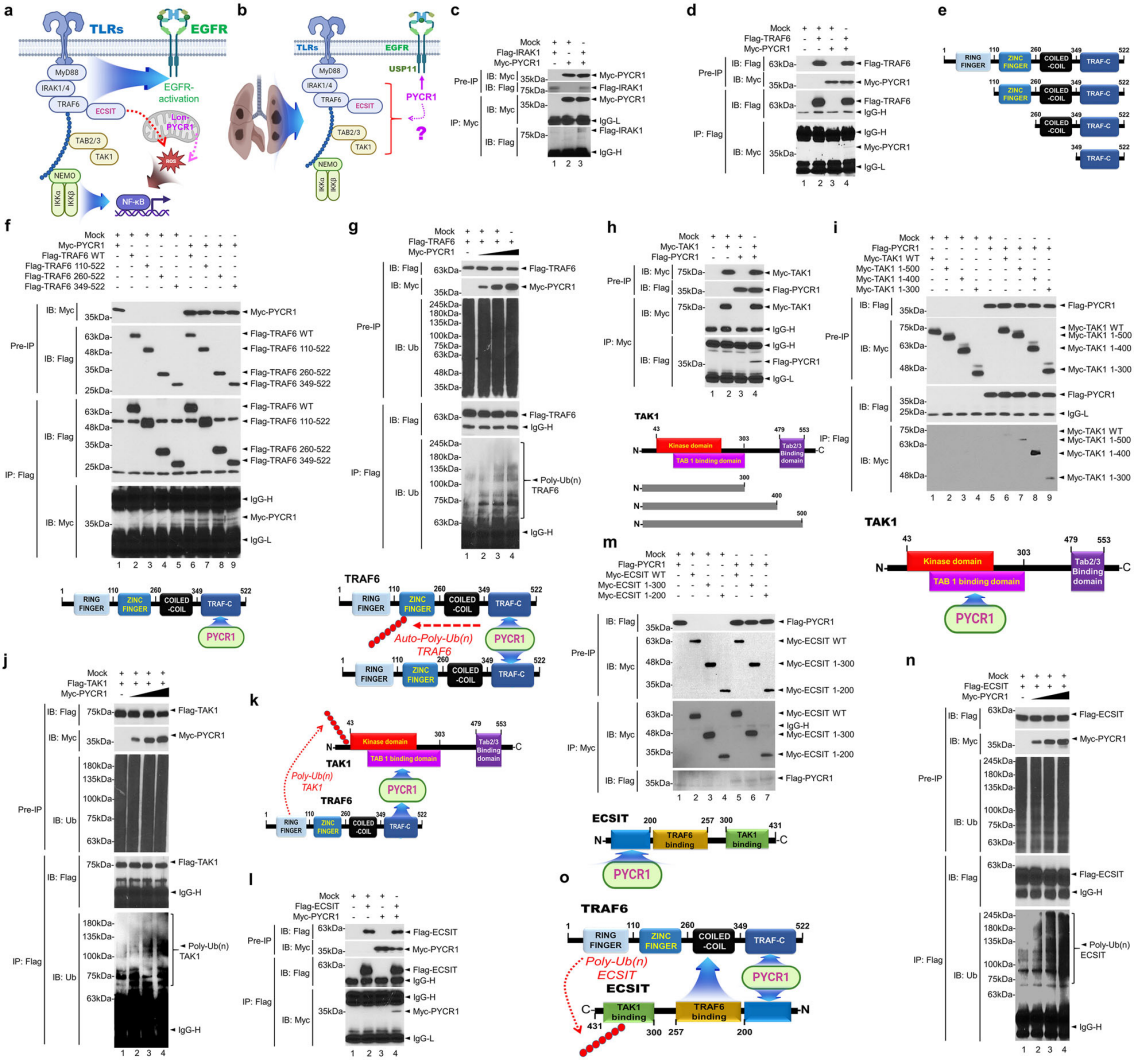
PYCR1 regulates TLR-mediated signaling through the interaction with TRAF6, TAK1 and ECSIT. **a**, **b** A possible model for the functional interaction between PYCR1 and EGFR or TLR signaling in lung cancer (**a**, role of Lon–PYCR1 in TLR signaling; **b**, proposed model of PYCR1 in EGFR and TLR signaling). **c** The HEK-293T cells were transiently transfected with mock (pcDNA, a control vector), Myc-PYCR1 or Flag-IRAK1, as indicated. An IP assay was performed with an anti-Myc antibody, followed by an immunoblotting (IB) assay with anti-Flag or anti-Myc antibodies. **d** The HEK-293T cells were transiently transfected with mock, Myc-PYCR1 or Flag-TRAF6, as indicated. An IP assay was performed with an anti-Flag antibody, followed by IB assay with anti-Flag or anti-Myc antibodies. **e** The truncated mutants of TRAF6 were illustrated. **f** The HEK-293T cells were transiently transfected with mock, Myc-PYCR1 or Flag-TRAF6 WT and its truncated mutants, as indicated. An IP assay was performed with an anti-Flag antibody, followed by an IB assay with anti-Flag or anti-Myc antibodies. Bottom: a schematic illustration showing that PYCR1 interacts with the TRAF-C domain of TRAF6. **g** The HEK-293T cells were transiently transfected with mock, Myc-PYCR1 or Flag-TRAF6, as indicated. IP assay was performed with anti-Flag antibodies, followed by an IB assay with anti-Flag, anti-Myc or anti-Ub antibodies. Bottom: a schematic model illustrates TRAF6 domains and their interaction with PYCR1 through the TRAF-C domain, enhancing TRAF6-mediated ubiquitination. **h** The HEK-293T cells were transiently transfected with mock, Flag-PYCR1 or Myc-TAK1, as indicated. An IP assay was performed with an anti-Myc antibody, followed by IB assay with anti-Flag or anti-Myc antibodies. Bottom: the truncated mutants of TAK1 were illustrated. **i** The HEK-293T cells were transiently transfected with mock, Flag-PYCR1 or Myc-TAK1 WT and its truncated mutants, as indicated. An IP assay was performed with an anti-Flag antibody, followed by an IB assay with anti-Flag or anti-Myc antibodies. Bottom: a schematic illustration showing that PYCR1 interacts with the TAB1-binding domain of TAK1. **j** The HEK-293T cells were transiently transfected with mock, Myc-PYCR1 or Flag-TAK1, as indicated. An IP assay was performed with anti-Flag antibodies, followed by an IB assay with anti-Flag, anti-Myc or anti-Ub antibodies. **k** A schematic model illustrating that PYCR1 binds to the TAB1-binding domain of TAK1 and the TRAF-C domain of TRAF6, facilitating the enhancement of TRAF6-mediated ubiquitination of TAK1. **l** The HEK-293T cells were transiently transfected with mock, Myc-PYCR1 or Flag-ECSIT, as indicated. An IP assay was performed with anti-Flag antibodies, followed by an IB assay with anti-Flag or anti-Myc antibodies. **m** The HEK-293T cells were transiently transfected with pcDNA (control vector), Flag-PYCR1 or Myc-ECSIT WT and its truncated mutants, as indicated. An IP assay was performed with an anti-Myc antibody, followed by an IB assay with anti-Flag or anti-Myc antibodies. Bottom: a schematic illustration showing that PYCR1 interacts with the N-terminal domain of ECSIT. **n** The HEK-293T cells were transiently transfected with mock, Myc-PYCR1 or Flag-ECSIT, as indicated. An IP assay was performed with anti-Flag antibodies, followed by an IB assay with anti-Flag, anti-Myc or anti-Ub antibodies. **o** A schematic illustration showing that PYCR1 binds to the TRAF-C domain of TRAF6 and the N-terminal of ECSIT, enhancing TRAF6-mediated ubiquitination of ECSIT.

The activation of EGFR is functionally implicated with multiple TLRs, and its kinase activity is required for the TLR signaling cascade^40,41^. Furthermore, TLR1, TLR2, or TLR4 signaling induces mitochondrial ROS generation for the activation of NF-κB and bactericidal activity through TRAF6–ECSIT complex^17^. Importantly, mitochondrial oxidative stress by Lon-PYCR1 maintains an immunosuppressive tumor microenvironment that promotes cancer progression and metastasis^18^. Given that PYCR1 is functionally linked to EGFR signaling, we further hypothesized that PYCR1 plays a pivotal role in TLR signaling in lung cancer cells, as illustrated in Fig. 3a, b.

To test this, we investigated the biochemical interactions between PYCR1- and TLR-mediated signaling components. PYCR1 was found to interact with IRAK1 and TRAF6 (Fig. 3c, d). To identify the specific domain of TRAF6 involved in its interaction with PYCR1, truncated TRAF6 mutants were generated (Fig. 3e), and an IP assay was performed. PYCR1 interacted with the WT TRAF6 as well as its truncated mutants (Fig. 3f), indicating that the interaction occurs through the TRAF-C domain of TRAF6 (Fig. 3f, bottom). The autoubiquitination of TRAF6 is crucial for TLR-mediated NF-κB activation, as it facilitates TRAF6 dimerization. To determine whether PYCR1 affects TRAF6 ubiquitination, we assessed this interaction in a dose-dependent manner. PYCR1 remarkably enhanced TRAF6 ubiquitination (Fig. 3g), suggesting that PYCR1 promotes TRAF6 dimerization and subsequent autoubiquitination (Fig. 3g, bottom). Next, we explored whether PYCR1 influences the downstream molecules of TRAF6, including TAK1, ECSIT and TAB2. PYCR1 was shown to interact with TAK1 (Fig. 3h). To identify the binding domain of TAK1 involved in its interaction with PYCR1, truncated TAK1 mutants were generated (Fig. 3h, bottom). The IP assay revealed that PYCR1 interacts with TAK1 through the TAB1-binding domain of TAK1 (Fig. 3i). In addition, PYCR1 remarkably increased TAK1 ubiquitination in a dose-dependent manner (Fig. 3j), indicating that PYCR1 facilitates the formation of a TRAF6–TAK1 complex and promotes TAK1 ubiquitination (Fig. 3k). PYCR1 also interacted with ECSIT through its N-terminal domain (Fig. 3l, m) and enhanced ECSIT ubiquitination in a dose-dependent manner (Fig. 3n), suggesting that PYCR1 supports the formation of a TRAF6–ECSIT complex, thereby promoting ECSIT ubiquitination (Fig. 3o).

### PYCR1 promotes NF-κB activation by orchestrating the assembly of the TAK1–TAB2–TRAF6 complex in TLR signaling

**Fig. 4:**
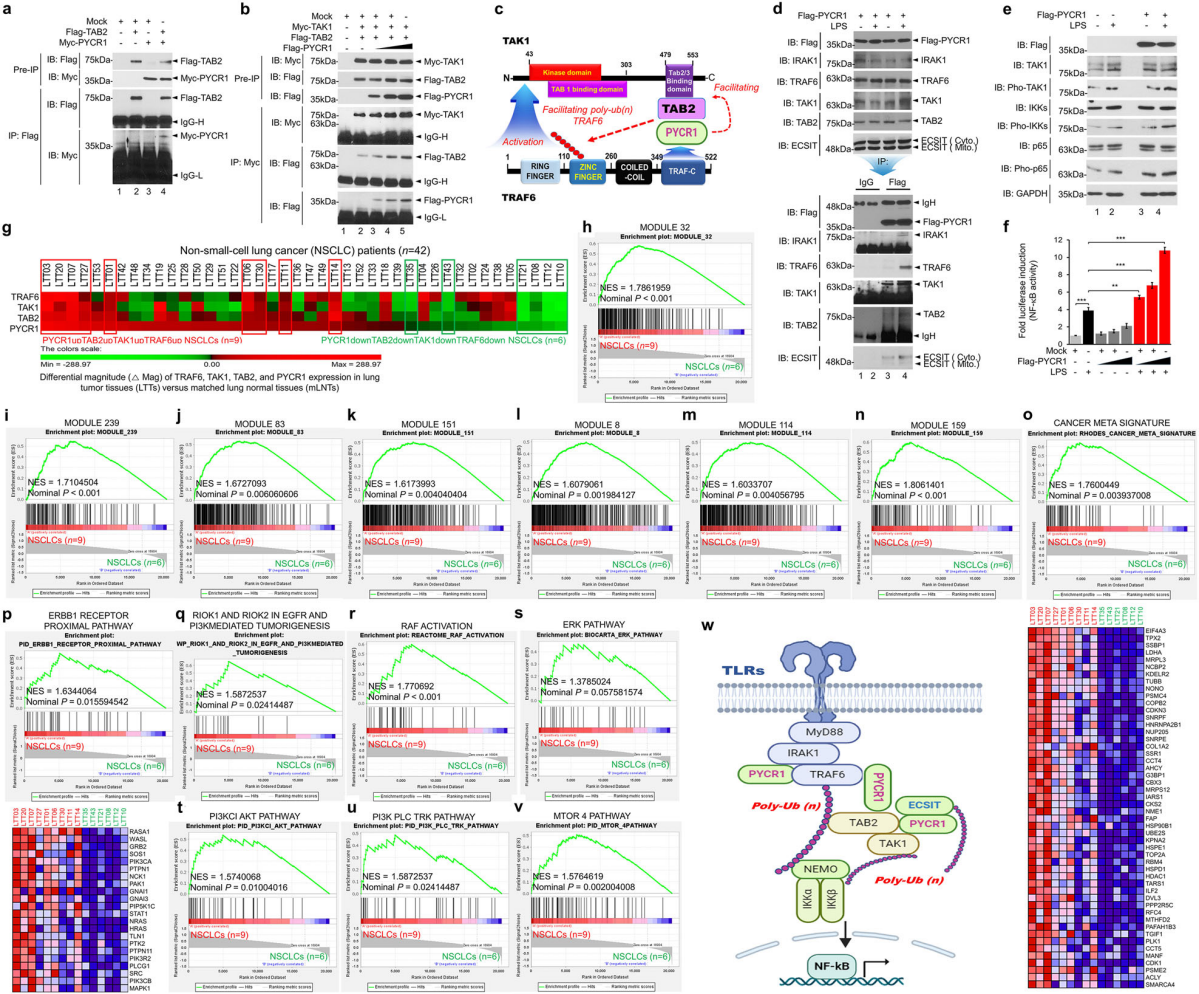
PYCR1 regulates the formation of TRAF6–TAB2–TAK1 complex and correlates their expressions in patients with NSCLC. **a** The HEK-293T cells were transiently transfected with mock (pcDNA, a control vector), Myc-PYCR1 or Flag-TAB2, as indicated. An IP assay was performed with an anti-Flag antibody, followed by an immunoblotting (IB) assay with anti-Flag or anti-Myc antibodies. **b** The HEK-293T cells were transiently transfected with mock, Flag-PYCR1, Myc-TAK1 or Flag-TAB2, as indicated. An IP assay was performed with an anti-Myc antibody, followed by an IB assay with anti-Flag or anti-Myc antibodies. **c** A schematic model illustrating the role of PYCR1 in the regulation of TRAF6-mediated signaling pathways. PYCR1 interacts with TAB2 and increases the interaction between TAK1 and TAB2. This complex enhances the TRAF6-mediated polyubiquitination of TAK1. **d** The A549 lung cancer cells were transiently transfected with Flag-PYCR1, and treated with or without LPS, as indicated. An IP assay was performed with anti-IgG or anti-Flag antibodies, followed by an IB assay with anti-Flag, anti-IRAK1, anti-TRAF6, anti-TAK1, anti-TAB2 or anti-ECSIT antibodies. **e** The H1299 cells were transfected with mock or Flag-PYCR1 and stimulated with or without LPS, as indicated. An IB assay was performed with anti-Flag, anti-TAK1, anti-pho-TAK1, anti-IKKs, anti-pho-IKKs, anti-p65, anti-pho-p65 and anti-GAPDH antibodies. **f** The A549 cells were transfected with mock or different concentrations of Flag-PYCR1, as indicated. The cells were stimulated with or without LPS for 6 h, as indicated. An NF-κB dual-luciferase assay was performed. The results are presented as mean ± s.d. (*n* = 5). **P < 0.01, ***P < 0.001. **g**, Based on the differential magnitude of PYCR1, TAB2, TAK1 and TRAF6 expression, 42 patients with NSCLC were categorized into two groups: those with upregulated PYCR1, TAB2, TAK1 and TRAF6 (*n* = 9, red boxes) and those with downregulated PYCR1, TAB2, TAK1 and TRAF6 (*n* = 6, green boxes). **h**–**v** The GSEA was conducted between the nine patients with upregulated PYCR1, TAB2, TAK1 and TRAF6 (PYCR1^up^TAB2^up^TAK1^up^TRAF6^up^ NSCLCs, red) and the six patients with downregulated PYCR1, TAB2, TAK1 and TRAF6 (PYCR1^down^TAB2^down^TAK1^down^TRAF6^down^ NSCLCs, green); the gene sets related to cancer modules (in **h**–**n**) cancer meta signature (**o**) and EGFR signaling (in **p**–**v**) are represented (Module 32 (**h**) Module 239 (**i**) Module 83 (**j**) Module 151 (**k**) Module 8 (**l**) Module 114 (**m**) Module 159 (**n**) Cancer meta signature (**o**) ERBB1 receptor proximal pathway (**p**) RIOK1 and RIOK2 in EGFR and PI3K-mediated tumorigenesis (**q**) Raf activation (**r**); Erk pathway (**s**); PI3K AKT pathway (**t**) PI3K PLC TRK pathway (**u**); mTOR4 pathway (**v**)). Bottom: a heat map associated with the Cancer meta signature (in **o**) and the ERBB1 receptor proximal pathway (in **p**) are shown. The NES and nominal *P* value are indicated in the inner panel. **w** A schematic model of how PYCR1 regulates TLR-mediated signaling for the activation of NF-κB.

TAB2, a TAK1-binding protein, facilitates TRAF6 ubiquitination and supports the formation of the TRAF6–TAB2–TAK1 complex for NF-κB activation^42^. PYCR1 was found to interact with TAB2 (Fig. 4a) and enhance the formation of a TAK1–TAB2 complex in a dose-dependent manner (Fig. 4b). This interaction suggests that PYCR1 facilitates the assembly of a TAK1–TAB2–PYCR1–TRAF6 complex, driving TRAF6 ubiquitination and TAK1 activation (Fig. 4c). To validate these molecular interactions, semiendogenous IP assays were performed. PYCR1 interacted with endogenous IRAK1, TRAF6, TAK1, TAB2 and ECSIT, with interactions importantly enhanced following LPS stimulation (Fig. 4d). Functionally, the PYCR1 overexpression augmented the activation of TAK1, IKKs and p65 in response to LPS stimulation, along with the activation of NF-κB (Fig. 4e, f). Importantly, association analysis in patients with NSCLC showed that six cancer-related modules—module 239 (invasion and metastasis), module 83 (tumor growth and metastatic spread), module 151 (EMT and migratory capacity), module 8 (hot tumor immune microenvironment), module 114 (genomic instability and rapid proliferation) and module 159 (angiogenesis and EMT)—along with a cancer metasignature gene set were remarkably enriched in the TRAF6^up^TAK1^up^TAB2^up^PYCR1^up^ group (*n* = 9) compared with the TRAF6^down^TAK1^down^TAB2^down^PYCR1^down^ group (*n* = 6) (Fig. 4g–o and Supplementary Table 3). Moreover, the gene sets related to EGFR signaling were remarkably enriched in TRAF6^up^TAK1^up^TAB2^up^PYCR1^up^ patients (*n* = 9) (Fig. 4p–v). Taken together, as depicted in Fig. 4w, PYCR1 plays a pivotal role in nucleating the molecular associations of TLR-mediated signaling molecules—including IRAK1, TRAF6, ECSIT, TAB2 and TAK1—thereby promoting NF-κB activation.

### PYCR1 facilitates EGFR- and TLR-mediated signaling cascades

**Fig. 5:**
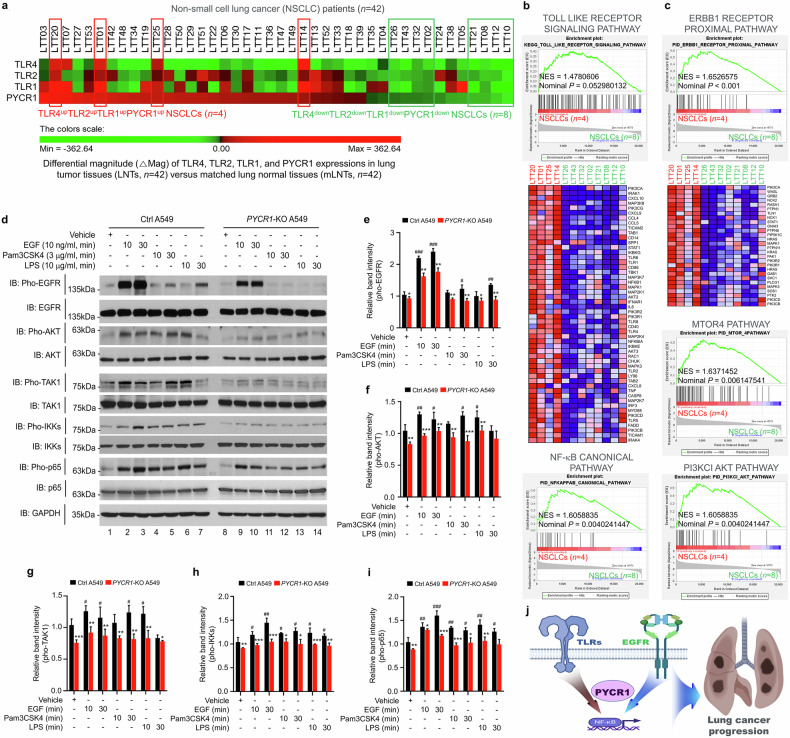
PYCR1 expression is associated with TLR4, TLR2 and TLR1 expression in patients with NSCLC. **a** Based on the differential magnitude of TLR4, TLR2, TLR1 and PYCR1 expression, 42 patients with NSCLC were categorized into two groups: those with TLR4^up^TLR2^up^TLR1^up^PYCR1^up^ (*n* = 4, red boxes) and those with TLR4^down^TLR2^down^TLR1^down^PYCR1^down^ (*n* = 8, green boxes). **b**, **c** A GSEA was conducted between the four patients with TLR4^up^TLR2^up^TLR1^up^PYCR1^up^ NSCLCs (red) and the eight patients with TLR4^down^TLR2^down^TLR1^down^PYCR1^down^ NSCLCs (green); the gene sets related to cancer progression are represented (TLR signaling pathway and NF-κB canonical pathway (**b**) and ERBB1 receptor proximal pathway, mTOR4 pathway and PI3KCI AKT pathway (**c**)). A heat map associated with the TLR signaling pathway (in **b**, bottom) and the ERBB1 receptor proximal pathway (in **c**, bottom) are shown. The NES and nominal *P* value are indicated in the inner section. **d**, The Ctrl A549 and *PYCR1*-KO A549 cells were treated with vehicle (DMSO, 0.1% v/v concentration), Pam3CSK4 (3 μg/ml), LPS (10 μg/ml) or EGF (10 ng/ml) for different times, as indicated. An immunoblotting (IB) assay was performed with anti-pho-EGFR, anti-EGFR, anti-pho-AKT, anti-AKT, anti-pho-TAK1, anti-TAK1, anti-pho-IKKs, anti-IKKs, anti-pho-p65, anti-p65 and anti-GAPDH antibodies. **e**–**i**, The band intensity of pho-EGFR (**e**) pho-AKT (**f**) pho-TAK1 (**g**) pho-IKKs (**h**) and pho-p65 (**i**) was analyzed with ImageJ Software. The error bars represent ± s.d. (*n* = 3). *P < 0.05, **P < 0.01, ***P < 0.001: ^#^*P* < 0.05, ^##^*P* < 0.01, ^###^*P* < 0.001; vehicle-treated cells versus EGF-, Pam3CSK4- and LPS-treated cells. **j** A schematic illustration showing that PYCR1 is associated with TLR and EGFR signaling pathways, promoting NF-κB activation and contributing to lung cancer progression.

Building on the biochemical evidence that PYCR1 regulates TLR-mediated signaling, we investigated the clinical association between PYCR1 expression and the expression of TLR4, TLR2 or TLR1 in patients with NSCLC. The patients were categorized into two groups: one group with upregulated expression of TLR4, TLR2, TLR1 and PYCR1 (*n* = 4), and another group with downregulated expression of these genes (*n* = 8) (Fig. 5a and Supplementary Table 4). The GSEA revealed that cancer-related gene modules were remarkably enriched in patients with upregulated TLR4, TLR2, TLR1 and PYCR1 (TLR4^up^TLR2^up^TLR1^up^PYCR1^up^) compared with patients with a downregulated expression of these markers (TLR4^down^TLR2^down^TLR1^down^PYCR1^down^) (Supplementary Fig. 5a–l). Importantly, the gene sets associated with TLR and EGFR signaling were highly enriched in patients with TLR4^up^TLR2^up^TLR1^up^PYCR1^up^ NSCLC (Fig. 5b, c and Supplementary Fig. 6a–d). These findings suggest a clinical correlation between PYCR1 expression and the activation of EGFR- and TLR-mediated signaling in NSCLC.

To explore the functional role of PYCR1 in EGFR- and TLR-mediated signaling, Ctrl A549 and *PYCR1*-KO A549 cells were treated with vehicle, EGF, Pam3CSK4 (a TLR1 and TLR2 agonist) or LPS (a TLR4 agonist) for various durations as indicated in Fig. 5d. In *PYCR1*-KO A549 cells treated with EGF, Pam3CSK4 or LPS, the levels of phosphorylated EGFR (pho-EGFR) and phosphorylated AKT (pho-AKT) were significantly reduced compared with Ctrl A549 cells (Fig. 5d–f). Moreover, the levels of phosphorylated TAK1 (pho-TAK1), IKKs (pho-IKKs) and p65 (pho-p65)—key mediators of TLR-induced NF-κB activation—were markedly decreased in *PYCR1*-KO A549 cells treated with EGF, Pam3CSK4 or LPS compared with Ctrl cells (Fig. 5d, g–i). These results demonstrate that PYCR1 plays a functional role in facilitating EGFR- and TLR-mediated signaling cascades, which are probably involved in EGFR- and TLR-driven lung cancer progression (Fig. 5j).

### PYCR1 promotes lung cancer progression induced by EGF and TLR stimulation

**Fig. 6:**
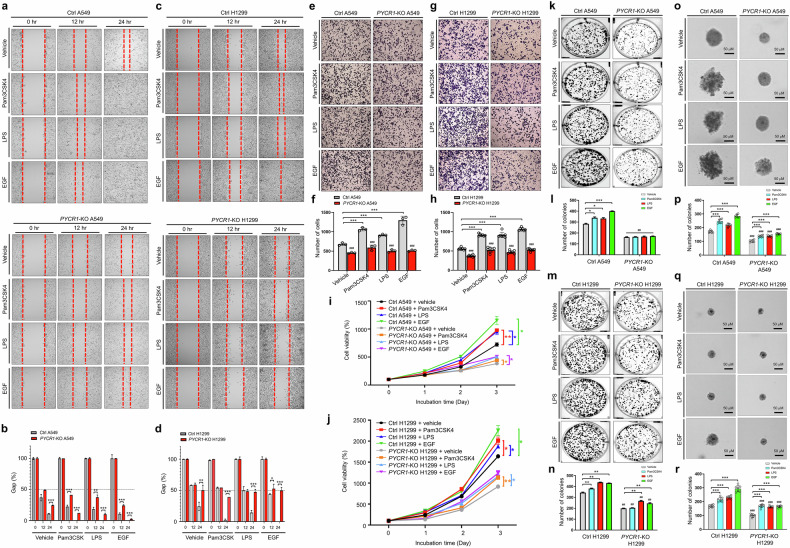
PYCR1 modulates EGFR- or TLR-mediated lung cancer migration, proliferation and colony formation. **a**–**d** A wound-healing assay was performed with Ctrl A549 and *PYCR1*-KO A549 cells (**a**, phase-contrast microscopy images; **b**, percentage of gap) or Ctrl H1299 and *PYCR1*-KO H1299 cells (**c**, phase-contrast microscopy images; **d**, percentage of gap). The cells were treated with vehicle (DMSO, 0.1% v/v concentration), Pam3CSK4 (3 µg/ml), LPS (10 µg/ml) or EGF (15 ng/ml). The residual gap between migrating cells from the opposing wound edge is expressed as a percentage of the initial scraped area (± s.d., *n* = 3 different plates). *P < 0.05, **P < 0.01, ***P < 0.001. **e**–**h** The Transwell migration assay was performed with Ctrl A549 and *PYCR1*-KO A549 cells (**e**, phase-contrast microscopy images; **f**, number of cells) or Ctrl H1299 and *PYCR1*-KO H1299 cells (**g**, phase-contrast microscopy images; **h**, number of cells). The cells were treated with vehicle (DMSO, 0.1% v/v concentration), Pam3CSK4 (3 µg/ml), LPS (3 µg/ml) or EGF (15 ng/ml). The absolute number of migrated cells is presented as the mean ± s.d. (in **f**, *n* = 3; in **h**, *n* = 7). ***P < 0.001. **i**, **j** An MTT assay was performed in Ctrl A549 and *PYCR1*-KO A549 cells (**i**) or Ctrl H1299 and *PYCR1*-KO H1299 cells (**j**). The cells were treated with vehicle (DMSO, 0.1% v/v concentration), Pam3CSK4 (1 µg/ml), LPS (2.5 µg/ml) or EGF (10 ng/ml) for different time periods, as indicated. The results are presented as mean ± s.d. (*n* = 5). *P < 0.05, **P < 0.01. **k**–**n** An anchorage-dependent colony formation assay was performed in Ctrl A549 and *PYCR1*-KO A549 cells (**k**, phase-contrast microscopy images; **l**, number of colonies) or Ctrl H1299 and *PYCR1*-KO H1299 cells (**m**, phase-contrast microscopy images; **n**, number of colonies). The cells were treated with vehicle (DMSO, 0.1% v/v concentration), Pam3CSK4 (3 µg/ml), LPS (5 µg/ml) or EGF (20 ng/ml). The results are presented as mean ± s.d. (*n* = 5). *P < 0.05, **P < 0.01, ***P < 0.001. **o**–**r**, An anchorage-independent soft agar colony formation assay was performed in Ctrl A549 and *PYCR1*-KO A549 cells (**o**, phase-contrast microscopy images; **p**, number of colonies) or Ctrl H1299 and *PYCR1*-KO H1299 cells (**q**, phase-contrast microscopy images; **r**, number of colonies). The cells were treated with vehicle (DMSO, 0.1% v/v concentration), Pam3CSK4 (1 µg/ml), LPS (10 µg/ml) or EGF (20 ng/ml). The results are presented as mean ± s.d. (*n* = 5; scale bar, 50 μm). ***P < 0.001.

Cancer progression is a systemic process characterized by the worsening or spread of cancer within the body, encompassing proliferation, migration and tumor formation^43,44^. Given the established role of PYCR1 in regulating EGFR- and TLR-mediated signaling, we investigated whether PYCR1 influences lung cancer progression in response to EGF and TLR agonists. We observed that the migratory ability of lung cancer cells, assessed using wound-healing and Transwell assays, was significantly reduced in *PYCR1*-KO A549 and *PYCR1*-KO H1299 cells treated with EGF or TLR agonists compared with Ctrl cells (Fig. 6a–h). In addition, cell proliferation was markedly decreased in *PYCR1*-KO A549 and *PYCR1*-KO H1299 cells upon stimulation with EGF or TLR agonists compared with Ctrl cells (Fig. 6i, j). Colony formation ability, assessed using both anchorage-dependent and anchorage-independent assays, was also significantly diminished in *PYCR1*-KO lung cancer cells treated with EGF or TLR agonists (Fig. 6k–n, o–r, anchorage-dependent assays and anchorage-independent assays, respectively), indicating that PYCR1 enhances lung cancer cell proliferation, migration and colony formation in response to EGF or TLR stimulation.

**Fig. 7:**
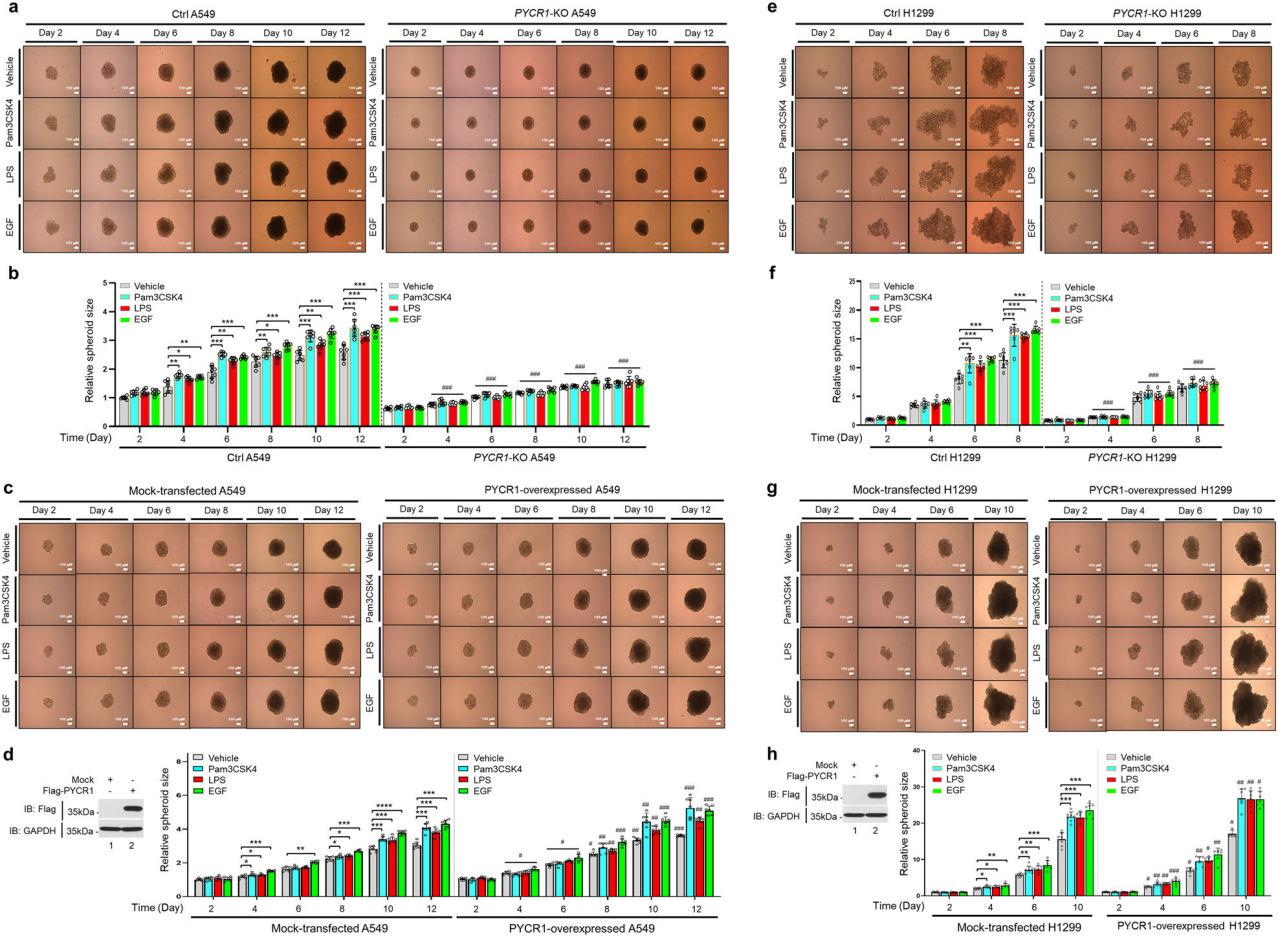
Functional role of PYCR1 expression in EGFR- or TLR-induced 3D tumor spheroid formation. **a**, **b** The spheroid formation and growth were evaluated using phase-contrast microscopy (scale bar, 100 μm) (**a**, phase-contrast microscopy images). The Ctrl A549 and *PYCR1*-KO A549 cells were seeded and incubated at 37 °C for 48 h to allow 3D spheroid formation. The spheroids were treated with vehicle (DMSO, 0.1% v/v concentration), Pam3CSK4 (2 µg/ml), LPS (5 µg/ml) or EGF (10 ng/ml) for different time periods, as indicated. The size of the spheroids was assessed using ImageJ Software. The error bars represent ± s.d. (*n* = 7) (in **b**, spheroid sizes). *P < 0.05, **P < 0.01, ***P < 0.001: ^###^*P* < 0.001; Ctrl A549 versus *PYCR1*-KO A549. **c**, **d** The spheroid formation and growth were evaluated using phase-contrast microscopy (scale bar, 100 μm) (**c**, phase-contrast microscopy images), and the Flag-PYCR1 expression was confirmed by western blotting using anti-Flag or anti-GAPDH as a loading control (**d**, left). The WT A549 cells were transfected with mock (a control vector) or Flag-PYCR1. The spheroids were treated with vehicle, Pam3CSK4, LPS or EGF for different time periods, as indicated. The size of the spheroids was assessed using ImageJ Software. The error bars represent ± s.d (*n* = 7) (in **d**, spheroid sizes). *P < 0.05, **P < 0.01, ***P < 0.001: ^#^*P* < 0.05, ^##^*P* < 0.01, ^###^*P* < 0.001, mock-transfected A549 versus PYCR1-overexpressed A549. **e**, **f** The spheroid formation and growth were evaluated using phase-contrast microscopy (scale bar, 100 μm) (**e**, phase-contrast microscopy images). The Ctrl H1299 and *PYCR1*-KO H1299 cells were seeded and incubated at 37 °C for 48 h to allow 3D spheroid formation. The spheroids were treated with vehicle, Pam3CSK4, LPS or EGF for different time periods, as indicated. The size of the spheroids was assessed using ImageJ Software. The error bars represent ± s.d. (*n* = 7) (in **f**, spheroid sizes). **P < 0.01, ***P < 0.001: ^###^*P* < 0.001, Ctrl H1299 versus *PYCR1*-KO H1299. **g**, **h** The spheroid formation and growth were evaluated using phase-contrast microscopy (scale bar, 100 μm) (**g**, phase-contrast microscopy images) and the Flag-PYCR1 expression was confirmed by western blotting using anti-Flag or anti-GAPDH as a loading control (**h**, left). The WT H1299 cells were transfected with mock or Flag-PYCR1. The spheroids were treated with vehicle, Pam3CSK4, LPS or EGF for different time periods, as indicated. The size of the spheroids was assessed using ImageJ Software. The error bars represent ± s.d. (*n* = 7) (in **h**, spheroid sizes). *P < 0.05, **P < 0.01, ***P < 0.001: ^#^*P* < 0.05, ^##^*P* < 0.01, ^###^*P* < 0.001, Mock-transfected H1299 versus PYCR1-overexpressed H1299.

We further examined the role of PYCR1 in 3D tumor spheroid formation under EGF and TLR agonist treatment. The tumor spheroid size increased in Ctrl A549 cells treated with EGF or TLR agonists, whereas spheroid size was significantly reduced in *PYCR1*-KO A549 cells treated under the same conditions (Fig. 7a, b). To verify the functional significance of PYCR1, we transfected Ctrl A549 cells with either a mock control vector or a Flag-PYCR1 overexpression vector and evaluated the spheroid formation. The PYCR1-overexpressing A549 cells treated with EGF or TLR agonists exhibited significantly larger tumor spheroids compared with mock-transfected cells (Fig. 7c, d). Consistent results were observed in H1299 cells, where *PYCR1*-KO H1299 cells treated with EGF or TLR agonists showed smaller spheroids compared with Ctrl H1299 cells (Fig. 7e, f). Conversely, the PYCR1 overexpression in H1299 cells resulted in significantly increased spheroid size under EGF or TLR stimulation compared with mock-transfected cells (Fig. 7g, h). These findings collectively suggest that PYCR1 promotes lung cancer progression by enhancing proliferation, migration, colony formation and tumor spheroid growth in response to EGF and TLR stimulation.

### PYCR1-IN-1, a PYCR1 inhibitor, suppresses EGFR- and TLR-induced 3D tumor spheroid growth in lung cancer cells

**Fig. 8:**
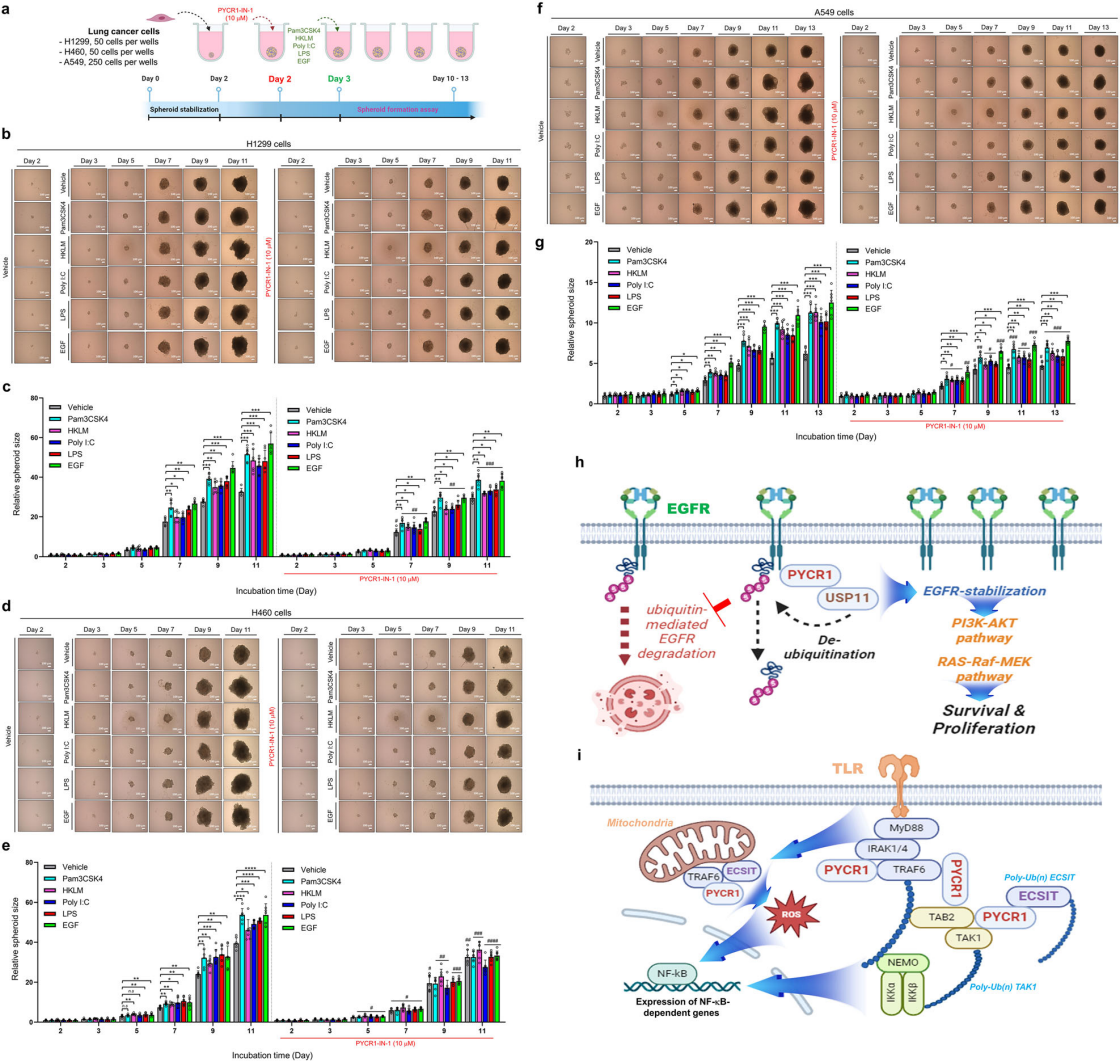
PYCR1 inhibitor, PYCR1-IN-1 attenuates EGFR- and TLRs-induced 3D tumor spheroid formation. **a** An experimental protocol for the evaluation of therapeutic effect of a PYCR1 inhibitor, PYCR1-IN-1, in WT H1299 (50 cells per well), H460 (50 cells per well) and A549 (250 cells per well) cells. After the stabilization of tumor spheroids for 2 days, the spheroids were treated with 10 μM PYCR1-IN-1, treated with vehicle (DMSO, 0.1% v/v concentration), Pam3CSK4 (3 μg/ml), HKLM (10^7^/ml), Poly(I:C) (5 μg/ml), LPS (5 μg/ml) or EGF (10 ng/ml) and incubated for different times, as indicated. **b**, **c** The H1299-derived tumor spheroid formation and growth were evaluated using phase-contrast microscopy (scale bar, 100 μm) (**b**, phase-contrast microscopy images). The spheroid sizes were measured using ImageJ Software. The error bars represent ± s.d. (*n* = 7) (in **c**, spheroid sizes). *P < 0.05, **P < 0.01, ***P < 0.001: ^#^*P* < 0.05, ^##^*P* < 0.01, ^###^*P* < 0.001, H1299 spheroids treated without PYCR1-IN-1 versus H1299 spheroids treated with PYCR1-IN-1. **d**, **e** H460-derived tumor spheroid formation and growth were evaluated using phase-contrast microscopy (scale bar, 100 μm) (**d**, phase-contrast microscopy images). The spheroid sizes were measured using ImageJ Software. The error bars represent ± s.d. (*n* = 7) (in **e**, spheroid sizes). *P < 0.05, **P < 0.01, ***P < 0.001, ****P < 0.0001: ^#^*P* < 0.05, ^##^*P* < 0.01, ^###^*P* < 0.001, ^####^*P* < 0.0001, H460 spheroids treated without PYCR1-IN-1 versus H460 spheroids treated with PYCR1-IN-1. **f**, **g** A549-derived tumor spheroid formation and growth were evaluated using phase-contrast microscopy (scale bar, 100 μm) (**f**, phase-contrast microscopy images). The spheroid sizes were measured using ImageJ Software. The error bars represent ± s.d. (*n* = 7) (in **g**, spheroid sizes). *P < 0.05, **P < 0.01, ***P < 0.001: ^#^*P* < 0.05, ^##^*P* < 0.01, ^###^*P* < 0.001, A549 spheroids treated without PYCR1-IN-1 versus A549 spheroids treated with PYCR1-IN-1. **h**, **i** A schematic representation of the therapeutic target in EGFR- (**h**) or TLR-driven (**i**) lung cancer progression. PYCR1 interacts with USP11, resulting in the deubiquitination and subsequent stabilization of EGFR in **h**. PYCR1 interacts with TLR-mediated signaling molecules, such as IRAK1, TRAF6, TAB2 and ECSIT, leading to increased ROS production and enhanced NF-κB activity in **i**.

Having established the role of PYCR1 in lung cancer progression, we investigated its potential as a therapeutic target against EGFR- and TLR-induced tumor formation. To this end, we utilized PYCR1-IN-1, a chemical inhibitor of PYCR1, and conducted preliminary assessments of its effectiveness in reducing the growth of 3D tumor spheroids derived from lung cancer cells to determine the optimal concentration. WT A549 and WT H1299 lung cancer cells were treated with PYCR1-IN-1 at concentrations ranging from 3 μM to 81 μM (Supplementary Fig. 7a–d). Treatment with PYCR1-IN-1 significantly reduced spheroid size in both WT A549 and WT H1299 cells at concentrations between 9 μM and 81 μM compared with vehicle-treated controls (Supplementary Fig. 7a–d). Based on these results, we examined the effects of PYCR1-IN-1 at a concentration of 10 μM, which induced approximately 20% cytotoxicity in lung cancer cells (Supplementary Fig. 7b, d). Interestingly, treatment with PYCR1-IN-1 markedly reduced cell proliferation, migration and anchorage-dependent colony formation in two additional lung cancer cell lines, H460 and H358, in response to TLR agonists and EGF (Supplementary Fig. 8a–e, vehicle versus PYCR1-IN-1 in H460 and Supplementary Fig. 9a–e, vehicle versus PYCR1-IN-1 in H358). A total of four different lung cancer cells—H1299, H460, A549 and H358 cells—were seeded, incubated to stabilize tumor spheroids for 48 h and treated with vehicle or 10 μM PYCR1-IN-1, following treatments of vehicle, TLR agonist or EGF, as illustrated in Fig. 8a. The EGF or TLR agonist treatments significantly increased spheroid size in all for cell lines in the absence of PYCR1-IN-1 compared with vehicle-treated controls (Fig. 8b–g and Supplementary Fig. 10a,b). Importantly, treatment with 10 μM PYCR1-IN-1 significantly reduced the size of EGFR- or TLR-induced tumor spheroids in H1299, H460, A549, H358 cells compared with cells treated with EGF or TLR agonists alone (Fig. 8b–g and Supplementary Fig. 10a,b). Furthermore, similar results could be observed in two EGFR mutant lung cancer cells, H1975 (EGFR mutant in L858R and T790M) and HCC827 (an exon 19 deletion mutant). The treatment with PYCR1-IN-1 markedly reduced the cell proliferation and the tumor spheroid size induced by the EGFR or TLR agonist in H1975 and HCC827 cells compared with cells treated with EGF or TLR agonists alone (Supplementary Fig. 11a–c, vehicle versus PYCR1-IN-1 in H1975, and Supplementary Fig. 12a–c, vehicle versus PYCR1-IN-1 in HCC827). These findings suggest that PYCR1 is a promising therapeutic target for intervening in EGFR- and TLR-induced lung cancer progression.

## Discussion

This study underscores the pivotal role of PYCR1 in driving lung cancer progression through its involvement in EGFR and TLR signaling pathways—two key regulators of tumor proliferation, migration and metastasis^9,10,14,15^. Our findings reveal that PYCR1 is significantly upregulated in patient-derived NSCLC tissues compared with their matched lung normal tissues, and its expression correlates with the activation of oncogenic pathways and in vivo tumorigenicity. Importantly, we provide molecular and cellular evidence demonstrating that PYCR1 is functionally implicated in EGFR- and TLR-driven lung cancer progression, highlighting its potential as both a biomarker and a therapeutic target for lung cancer.

Previous research has established that PYCR1 is involved in EGFR signaling pathways^45^. PYCR1 interacts with EGFR, activating the PI3K–AKT pathway to enhance aerobic glycolysis and support cancer cell proliferation in bladder cancer^45^. In addition, PYCR1 knockdown has been reported to inhibit proliferation, migration and invasion through the JAK–STAT signaling pathway in lung adenocarcinoma^46^. Despite these findings, the precise molecular mechanism by which PYCR1 regulates EGFR signaling has remained unexplored. In this study, we found that PYCR1 interacts with EGFR and USP11, leading to the deubiquitination and stabilization of EGFR, as illustrated in Fig. 8h. Functionally, *PYCR1*-KO lung cancer cells showed a significant reduction in cancer progression in response to EGF stimulation. Notably, treatment with the PYCR1 inhibitor, PYCR1-IN-1, suppressed EGFR-driven 3D tumor spheroid formation, further demonstrating the functional relevance of PYCR1 in EGFR signaling.

Several studies have suggested that PYCR1 may also play a role in TLR-related signaling pathway^18^. First, PYCR1 knockdown has been shown to inhibit proliferation, drug resistance and EMT by affecting the STAT3-mediated p38 MAPK and NF-κB pathways in colorectal cancer cells^46,47^. Second, mitochondrial oxidative stress induced by Lon-PYCR1 has been reported to maintain an immunosuppressive tumor microenvironment that promotes cancer progression and metastasis^18^. Furthermore, TLR activation is known to trigger ROS production, leading to the activation of NF-κB, IFN-regulatory factor 3 and STAT1-mediated innate immune responses^17,48–50^. Given the critical roles of NF-κB and ROS in TLR pathways^17,18,47–50^, we hypothesized that PYCR1 might be functionally implicated in TLR-mediated signaling.

Through biochemical analyses, we discovered that PYCR1 interacts with key TLR pathway components, including IRAK1, TRAF6, ECSIT, TAB2 and TAK1. Specifically, PYCR1 enhances the ubiquitination of TRAF6, ECSIT and TAK1, facilitating the activation of NF-κB signaling, as illustrated in Fig. 8i. Functionally, we observed that lung cancer cell migration, proliferation, colony formation and 3D tumor spheroid formation induced by TLR agonists were markedly attenuated following PYCR1 depletion. Furthermore, a PYCR1 chemical inhibitor, PYCR1-IN-1, significantly suppressed the 3D tumor spheroid formation driven by both TLR and EGFR signaling.

In conclusion, this study identifies PYCR1 as a central regulator of EGFR and TLR signaling pathways in lung cancer, as illustrated in Fig. 8h,i. PYCR1 stabilizes EGFR by facilitating its deubiquitination and simultaneously promotes TLR-mediated NF-κB activation, demonstrating its multifaceted role in tumor progression. The therapeutic inhibition of PYCR1, particularly using PYCR1-IN-1, highlights a promising strategy for targeting lung cancer driven by hyperactive EGFR and TLR signaling. Future studies should investigate the potential synergy between PYCR1 inhibitors and existing EGFR-targeted therapies or TLR inhibitors, as such combinations could enhance treatment efficacy and overcome resistance mechanisms. Moreover, elucidating the broader molecular interactions of PYCR1 across different oncogenic contexts may reveal its role in other cancer types. Finally, the clinical evaluation of PYCR1 inhibitors, including PYCR1-IN-1, will be essential to translate these findings into effective therapeutic strategies for patients with NSCLC.

## Supplementary information

Supplementary Information

## Data Availability

The data that support the findings of this study are available from the corresponding authors upon reasonable request.
