# Supplementary material for: PYCR1 drives lung cancer progression through functional interactions with EGFR and TLR signaling pathways
Source: Exp Mol Med. 2025 Nov 18;57(11):2559–73. doi: 10.1038/s12276-025-01577-z (PMC12686397; doi:10.1038/s12276-025-01577-z)
Supplement: Supplementary file 1 — Supplementary Information [file 12276_2025_1577_MOESM1_ESM.docx]

**Title:**

**PYCR1 drives lung cancer progression through functional interactions with EGFR and TLR signaling pathways**

Ji Hye Shin^1,#^, Ji Young Kim^1,#^, Mi-Jeong Kim^2,3,#^, Yeeun Kang^1,#^, Ha-Jeong Lee^1,#^, Bongkum Choi^4,5^, Ji Su Lee^1^, Dohee Kwon^5^, Seo Hyun Kim^1^, Yoolim Sung^1^, Duk-Hwan Kim^4^, Jae-Hyuck Shim^2,3^, Eunyoung Chun^6,*^, Ki-Young Lee^1,7,8,*^

Affiliations:

^1^Department of Immunology, Samsung Biomedical Research Institute, Sungkyunkwan University School of Medicine, Suwon, Republic of Korea

^2^Division of Rheumatology, Department of Medicine, University of Massachusetts Chan Medical School, Worcester, Massachusetts, USA

^3^Horae Gene Therapy Center, University of Massachusetts Chan Medical School, Worcester, Massachusetts, USA

^4^Department of Medicine, Sungkyunkwan University School of Medicine, Suwon, Republic of Korea

^5^Bioanalysis Center, GenNBio Inc., Seongnam, Republic of Korea

^6^Research and Development Center, CHA Vaccine Institute, Seongnam, Republic of Korea

^7^Samsung Medical Center, Department of Health Science and Technology, Samsung Advanced Institute for Health Science and Technology, Sungkyunkwan University School of Medicine, Seoul, Republic of Korea

^8^Department of Metabiohealth, Sungkyun Convergence Institute, Sungkyunkwan University, Suwon, Republic of Korea

^#^These authors contributed equally to this work

*Corresponding authors:

Ki-Young Lee, Department of Immunology and Samsung Biomedical Research Institute, Sungkyunkwan University School of Medicine, 2066 Seobu-ro, Jangan-gu, Suwon, Gyeonggi-do, 16419 Korea. Tel: +82-31-299-6225; Fax: +82-31-299-6229; E-mail: thylee@skku.edu.

Eunyoung Chun, Research and Development Center, CHA Vaccine Institute, 560 Dunchon-daero, Jungwon-gu, Seongnam-si, Gyeonggi-do, 13230 Korea. Tel: +82-31-881-7347; Fax: +82-31-737-8210; E-mail: [chun.eunyoung@gmail.com](mailto:chun.eunyoung@gmail.com).

**Supplementary Information**

**Contents:**

**MATERIALS AND METHODS**

**Supplementary Figures**

**Supplementary Figure 1. Association of PYCR1 expression in NSCLC patients.**

**Supplementary Figure 2.** **GSEA between PYCR1^up^ NSCLC patients (*n =* 29) and PYCR1^down^ NSCLC patients (*n =* 13).**

**Supplementary Figure 3. Attenuation of lung cancer cell viability in *PYCR1*-Knockout (*PYCR1*-KO) lung cancer cells.**

**Supplementary Figure 4. GSEA between PYCR1^up^ EGFR^up^ NSCLC patients (*n =* 20) and PYCR1^down^ EGFR^down^ NSCLC patients (*n =* 3).**

**Supplementary Figure 5. GSEA between the 4 patients with up-regulated TLR4, TLR2, TLR1, and PYCR1, and the 8 patients with down-regulated TLR4, TLR2, TLR1, and PYCR1.**

**Supplementary Figure 6. GSEA between the 4 patients with up-regulated TLR4, TLR2, TLR1, and PYCR1, and the 8 patients with down-regulated TLR4, TLR2, TLR1, and PYCR1.**

**Supplementary Figure 7.** **PYCR1-IN-1 Inhibits 3D tumor spheroid growth in lung cancer cells.**

**Supplementary Figure 8.** **PYCR1-IN-1 Inhibits cell proliferation, migration, and anchorage-dependent colony formation of H460 lung cancer cells in response to TLR agonists or EGF.**

**Supplementary Figure 9.** **PYCR1-IN-1 Inhibits cell proliferation, migration, and anchorage-dependent colony formation of H358 lung cancer cells in response to TLR agonists or EGF.**

**Supplementary Figure 10.** **PYCR1-IN-1 Inhibits 3D tumor spheroid growth in H358 lung cancer cells.**

**Supplementary Figure 11.** **PYCR1-IN-1 Inhibits cell proliferation and 3D tumor spheroid growth in EGFR mutant H1975 (L858R and T790M) lung cancer cells.**

**Supplementary Figure 12.** **PYCR1-IN-1 Inhibits cell proliferation and 3D tumor spheroid growth in EGFR mutant HCC827 (an exon 19 deletion) lung cancer cells.**

**Supplementary Tables**

**Supplementary Table 1.** Clinical characteristics of NSCLC patients (*n* = 42), and differential magnitude (ΔMag) of PYCR1 expression between lung tumor tissues (LTTs, *n* = 42) and matched lung normal tissues (mLNTs, *n* = 42).

**Supplementary Table 2.** Differential magnitude (ΔMag) of PYCR1 and EGFR expression between lung tumor tissues (LTTs, *n* = 42) and matched lung normal tissues (mLNTs, *n* = 42).

**Supplementary Table 3.** Differential magnitude (ΔMag) of TRAF6, TAK1, TAB2, and PYCR1 expression between lung tumor tissues (LTTs, *n* = 42) and matched lung normal tissues (mLNTs, *n* = 42).

**Supplementary Table 4**. Differential magnitude (ΔMag) of TLR4, TLR2, TLR1, and PYCR1 expression between lung tumor tissues (LTTs, *n* = 42) and matched lung normal tissues (mLNTs, *n* = 42).

**MATERIALS AND METHODS**

**MTT assay**

Ctrl A549, *PYCR1*-KO A549, Ctrl H1299, and *PYCR1*-KO H1299 cells were seeded into 96-well culture plates at a density of 700 cells/well and grown in a culture medium supplemented with 10% FBS for different time periods. H460, H358, H1975 (EGFR mutations / L858R and T790M), and HCC827 (EGFR mutation / an exon 19 deletion) cells were seeded into 96-well culture plates at density of 200 (H460 and H358) or 1000 (H1975 and HCC827) cells/well. The cells were treated with either vehicle (0.1% DMSO) or PYCR1-IN-1 (10 µM), followed by stimulation with vehicle (0.1% DMSO), TLR agonists - Pam3CSK4 (3 µg/ml), HKLM (10^7^/ml), Poly I:C (5 µg/ml), LPS (5 µg/ml) - and EGF (10 ng/ml) for the indicated time periods. Cell viability was measured using an MTT reagent (Sigma-Aldrich, M5655) dissolved in PBS (1 mg/mL). On the measurement day, the medium was carefully replaced with dPBS-diluted MTT (1:10, 10% MTT) and incubated for 3 hours at 37 °C. After incubation, the medium was removed, and formazan crystals were dissolved in 100 µL of DMSO. MTT reduction was quantified by measuring absorbance at 595 nm using a Bio-Rad Model 680 microplate reader (Bio-Rad, CA, USA). Each test was repeated at least four times in quadruplicate.

**Transwell migration assay**

H460 and H358 cells were suspended in a culture medium (250 μL) and added to the upper compartment of a 24-well Transwell® chamber (8 μm pore; Corning, 3422). H460 and H358 cells and free culture medium (250 μL) were mixed and treated with either vehicle (0.1% DMSO) or PYCR1-IN-1 (10 µM), followed by stimulation with vehicle (0.1% DMSO), various TLR agonists - Pam3CSK4 (3 µg/ml), HKLM (10^7^/ml), Poly I:C (5 µg/ml), LPS (5 µg/ml) - and EGF (10 ng/ml), and then cells were incubated at 37 °C for 24 h. Migratory cells would pass through polycarbonate membranes and cling to the bottom side. Non-migratory cells would stay in the upper chamber. After removing non-migratory cells, migratory cells were fixed using 2.5 % glutaraldehyde (Sigma-Aldrich, G6257-100 mL) and then stained with 0.1% crystal violet (Sigma-Aldrich, C6158-50g).

**Anchorage-dependent colony formation assay**

H460 and H358 cells were harvested with trypsin-EDTA and resuspended in a singular form. Then 300 cells/well were plated into a 6-well plate and treated with either vehicle (0.1% DMSO) or PYCR1-IN-1 (10 µM), followed by stimulation with vehicle (0.1% DMSO), various TLR agonists - Pam3CSK4 (3 µg/ml), HKLM (10^7^/ml), Poly I:C (5 µg/ml), LPS (5 µg/ml) - and EGF (10 ng/ml). After incubation for 8 days, colonies were stained with 0.5% crystal violet (Sigma-Aldrich, C6158-50g) for 30 min at room temperature.

**Three-dimension (3D) spheroids formation assay**

Wild-Type (WT) A549 (250 cells per well) or WT H1299 (50 cells per well) were seeded into 96-well plates and incubated at 37 °C for 48 hours to allow the formation of 3D spheroids in culture. The spheroids were treated with either vehicle (DMSO, 0.1 % v/v concentration) or different concentrations of PYCR1-IN-1, ranging from 81 μM to 3 μM. Spheroid sizes were measured using ImageJ Software. Error bars represent ± SD (*n =* 5) from three independent experiments. H358 (250 cells per well), H1975 (EGFR mutations/L858R and T790M, 250 cells per well), or HCC827 (EGFR mutation/ an exon 19 deletion, 500 cells per well) were seeded into 96-well plates and incubated at 37 °C for 48 hours to allow the formation of 3D spheroids in culture. Spheroids were then treated with vehicle (DMSO, 0.1% v/v) or 10 µM PYCR1-IN-1. After 24 hours, spheroids were further treated with vehicle (0.1% DMSO), Pam3CSK4 (3 µg/mL), HKLM (10^7^/mL), Poly I:C (5 µg/mL), LPS (5 µg/mL), or EGF (10 ng/mL). Tumor spheroid formation and growth were evaluated using phase-contrast microscopy, and spheroid sizes were measured using ImageJ Software.

**Gene set enrichment analysis (GSEA)**

Different magnitudes of PYCR1, EGFR, TLR4, TLR2, and TLR1 expression were obtained from pre-processed microarray data between lung tumor tissues and matched lung normal tissues. Based on the expression of PYCR1, EGFR, TLR4, TLR2, and TLR1, 42 NSCLC patients were stratified into up-regulated patients and down-regulated patients, as indicated in Figure 1a, Figure S4a, Figure 4g, and Figure 5a. Genes showing significant differences, such as normalized enrichment score (NES) and nominal P-value were analyzed by GSEA (<http://www.gsea-msigdb.org/gsea/index.jsp>).

**Statistical analysis**

All data are expressed as mean ± SD (standard deviation). Statistical significance was determined by Student’s t-test using GraphPad Prism 5.0 (GraphPad Software, San Diego, CA, USA). *P*-values were marked as **P* < 0.05, ***P* < 0.01, ****P* < 0.001, *****P* < 0.0001, ^#^*P* < 0.05, ^##^*P* < 0.01, ^###^*P* < 0.001, ^####^*P* < 0.0001.

**
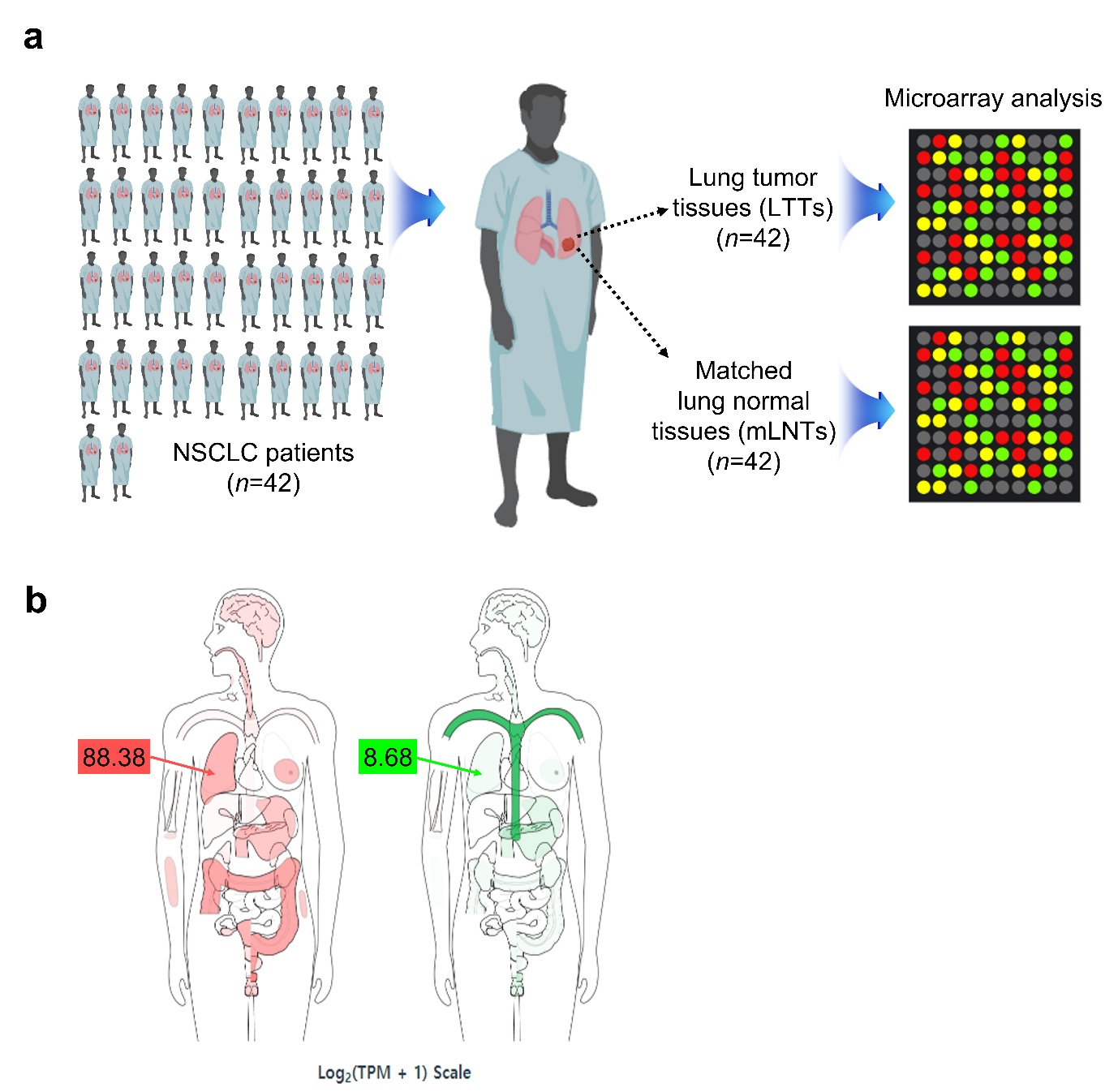
**

**Supplementary Figure 1. Association of PYCR1 expression in NSCLC patients.**

**a** Lung tumor tissues (LTTs) and matched lung normal tissues (mLNTs) were isolated from 42 NSCLC patients, and microarray analysis was performed. **b** A map of the human lung shows significantly higher expression of PYCR1 mRNA in tumors compared to non-tumor tissues (<http://gepia.cancer-pku.cn/detail.php?gene=PYCR>1).

**
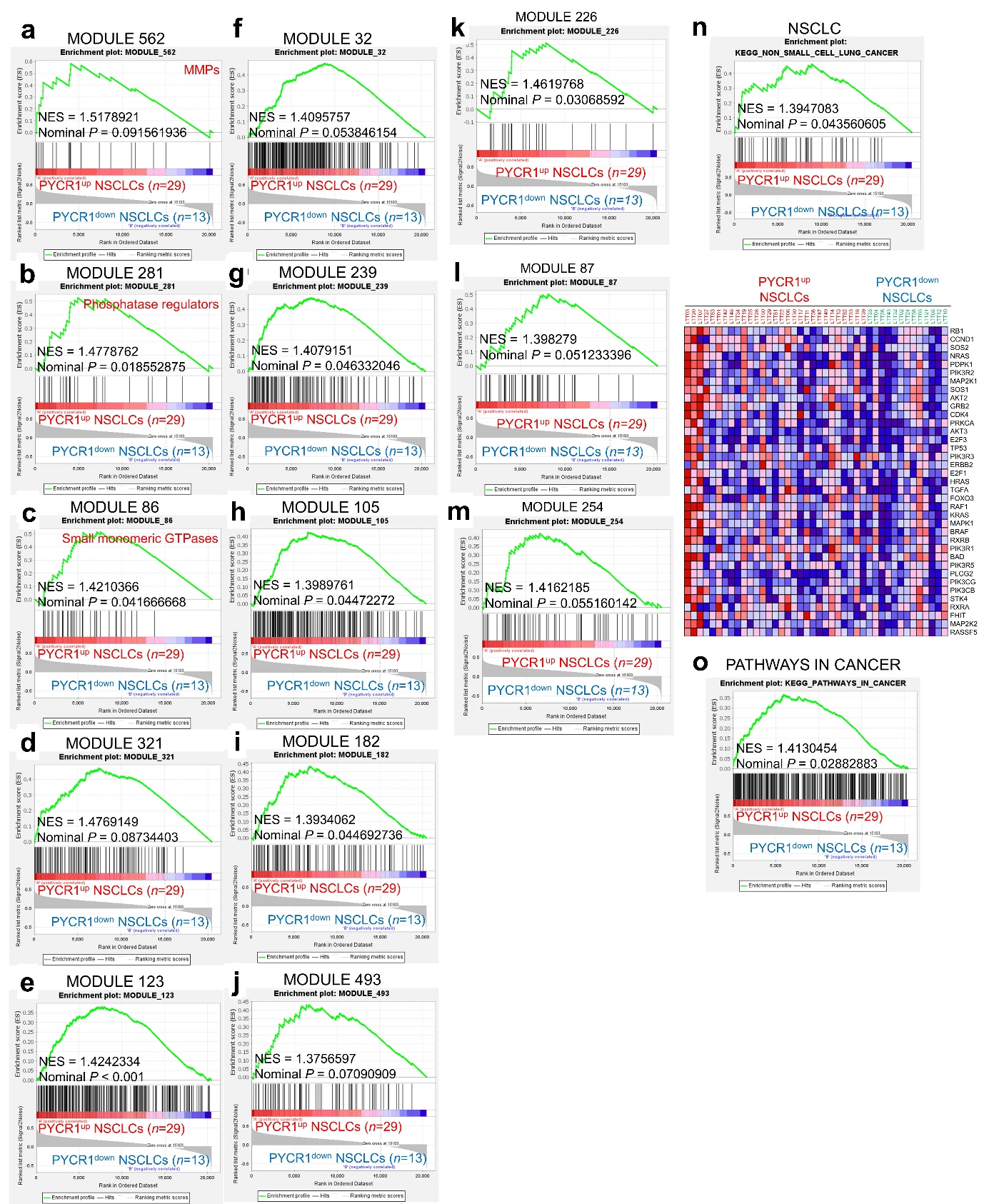
**

**Supplementary Figure 2.** **GSEA between PYCR1^up^ NSCLC patients (*n =* 29) and PYCR1^down^ NSCLC patients (*n =* 13).**

**a**-**o** Gene set enrichment analysis (GSEA, https://www.gsea-msigdb.org/gsea/index.jsp) was conducted between the 29 patients with up-regulated PYCR1 (PYCR1^up^ NSCLCs, red) and the 13 patients with down-regulated PYCR1 (PYCR1^down^ NSCLCs, blue). Gene sets related to cancer modules (**a-m**) and cancer progression (**n, o**) are represented (**a**, MODULE 562; **b**, MODULE 281; **c**, MODULE 86; **d**, MODULE 321; **e**, MODULE 123; **f**, MODULE 32; **g**, MODULE 239; **h**, MODULE 105; **i**, MODULE 182; **j**, MODULE 493; **k**, MODULE 226; **l**, MODULE 87; **m**, MODULE 254; **n**, NON-SMALL CELL LUNG CANCER; **o**, PATHWAYS IN CANCER). NES and nominal *P*-value are indicated in the inner panel. Heat-map associated in the NON-SMALL CELL LUNG CANCER is represented (**n**, down).

**
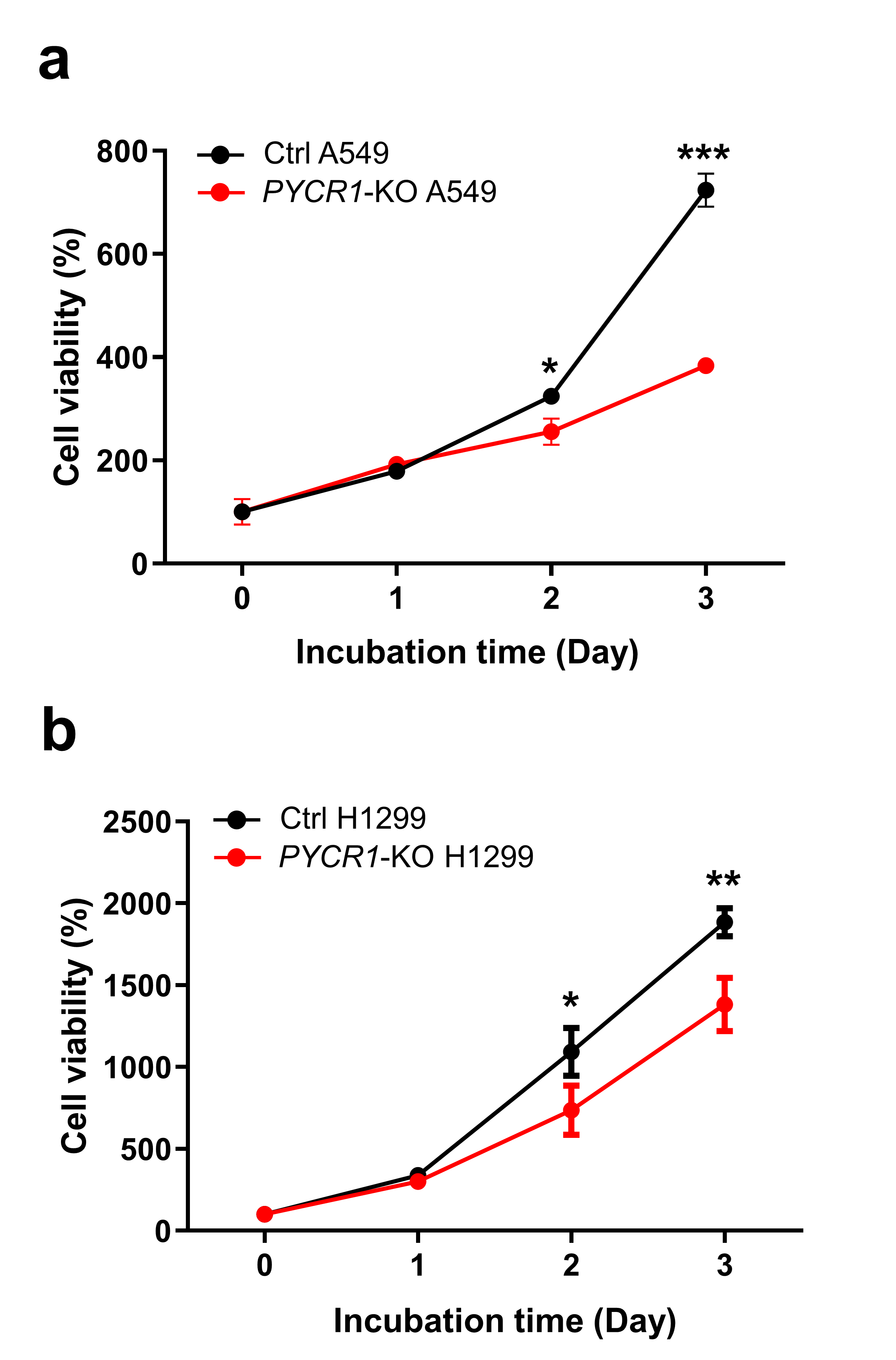
**

**Supplementary Figure 3. Attenuation of lung cancer cell viability in *PYCR1*-Knockout (*PYCR1*-KO) lung cancer cells.**

**a, b** MTT assay was performed in Ctrl A549 and *PYCR1*-KO A549 (**a**) or Ctrl H1299 and *PYCR1*-KO H1299 (**b**) cells for different time periods, as indicated. Results are presented as mean ± SD (*n* = 5). *, *P* < 0.05; **, *P* < 0.01; ***, *P* < 0.001.

**
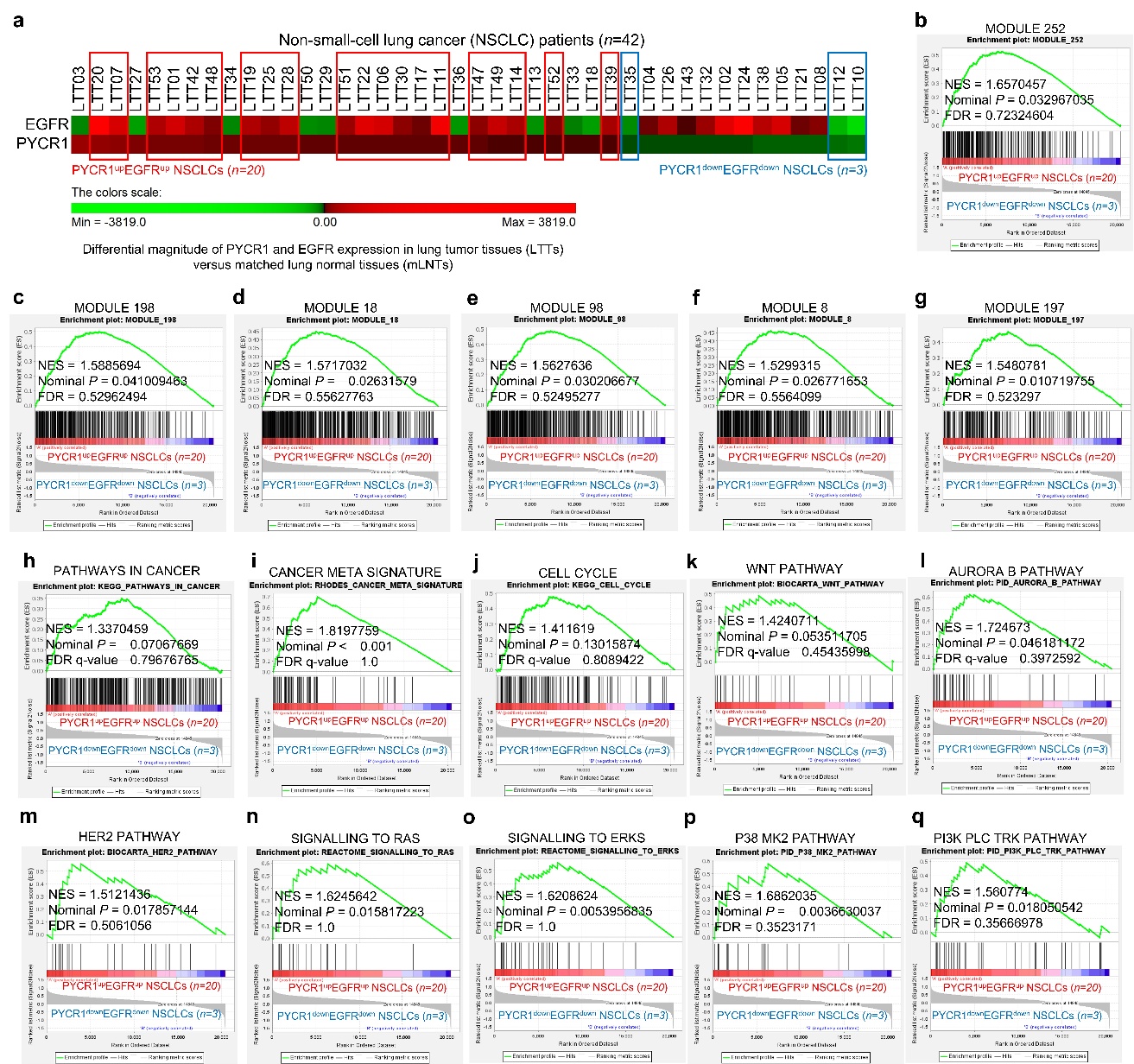
**

**Supplementary Figure 4. GSEA between PYCR1^up^ EGFR^up^ NSCLC patients (*n =* 20) and PYCR1^down^ EGFR^down^ NSCLC patients (*n =* 3).**

**a** Based on the differential magnitude of PYCR1 and EGFR expression, 42 NSCLC patients were categorized into two groups: those with up-regulated PYCR1 and EGFR (*n =* 20, red boxes) and those with down-regulated PYCR1 and EGFR (*n =* 3, blue boxes). (**b**-**q**) GSEA was conducted between the 20 patients with up-regulated PYCR1 and EGFR (PYCR1^up^EGFR^up^ NSCLCs, red) and the 3 patients with down-regulated PYCR1 and EGFR (PYCR1^down^EGFR^down^ NSCLCs, blue). Gene sets related to cancer modules (**b**-**g**), cancer progression (**h**-**l**) and EGFR-related signaling (**m**-**q**) are represented (**b**, MODULE 252; **c**, MODULE 198; **d**, MODULE 18; **e**, MODULE 98; **f**, MODULE 8; **g**, MODULE 197; **h**, PATHWAYS IN CANCER; **i**, CANCER META SIGNATURE; **j**, CELL CYCLE; **k**, WNT PATHWAY; **l**, AURORA B PATHWAY; **m**, HER2 PATHWAY; **n**, SIGNALING TO RAS; **o**, SIGNALING TO ERKS; **p**, P38 MK2 PATHWAY; **q**, PI3K PLC TRK PATHWAY). NES and nominal *P*-value are indicated in the inner panel.

**
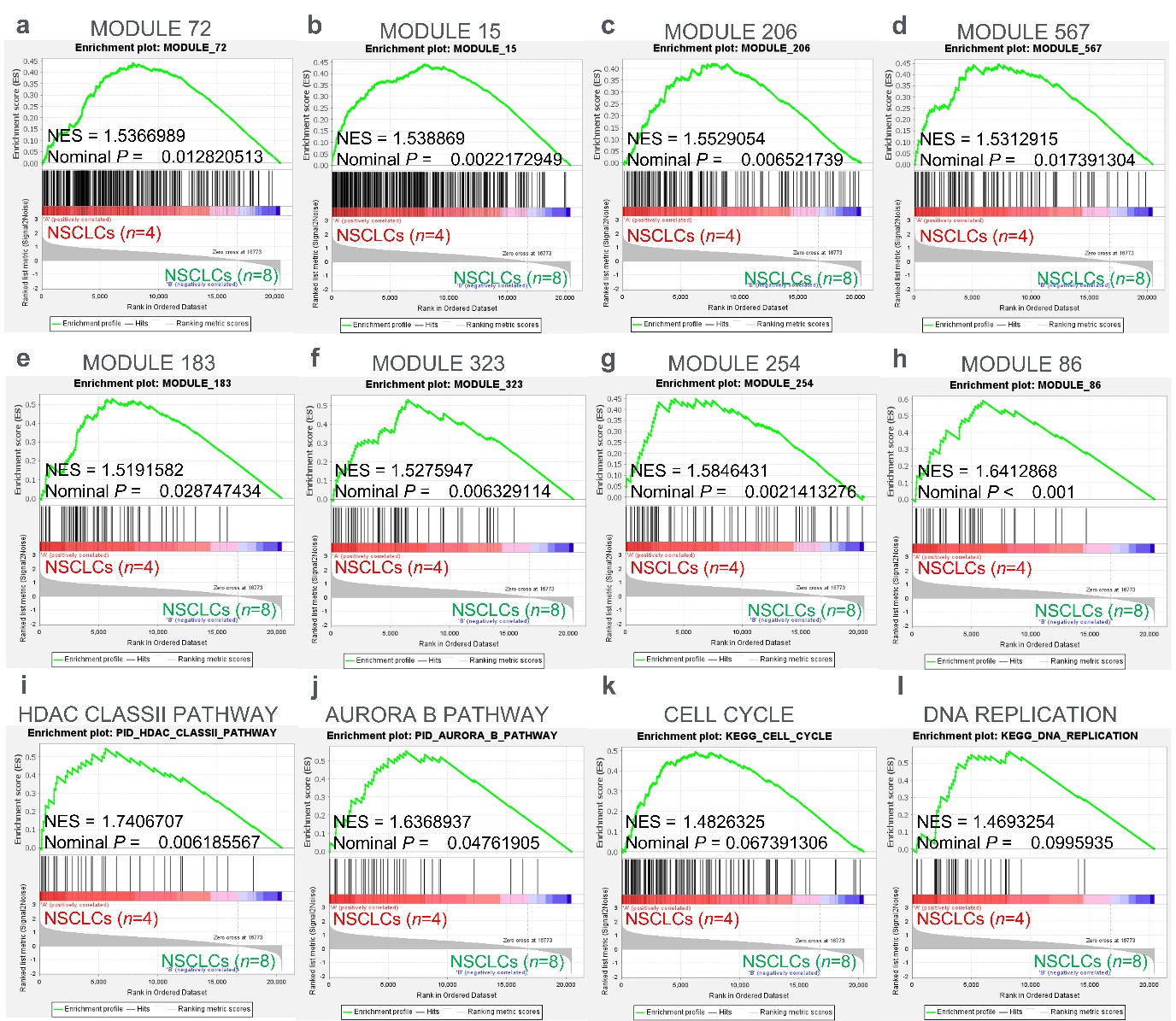
**

**Supplementary Figure 5. GSEA between the 4 patients with up-regulated TLR4, TLR2, TLR1, and PYCR1, and the 8 patients with down-regulated TLR4, TLR2, TLR1, and PYCR1.**

**a**-**l** GSEA was conducted between the 4 patients with up-regulated TLR4, TLR2, TLR1, and PYCR1 (TLR4^up^TLR2^up^TLR1^up^PYCR1^up^ NSCLCs, red) and the 8 patients with down-regulated TLR4, TLR2, TLR1, and PYCR1 (TLR4^down^TLR2^down^TLR1^down^PYCR1^down^ NSCLCs, green). Gene sets related to cancer modules (**a**-**h**) and cancer progression (**i**-**l**) are represented (**a**, MODULE 72; **b**, MODULE 15; **c**, MODULE 206; **d**, MODULE 567; **e**, MODULE 183; **f**, MODULE 323; **g**, MODULE 254; **h**, MODULE 86; **i**, HDAC CLASSII PATHWAY; **j**, AURORA B PATHWAY; **k**, CELL CYCLE; **l**, DNA REPLICATION). NES and nominal *P*-value are indicated in the inner panel.

**
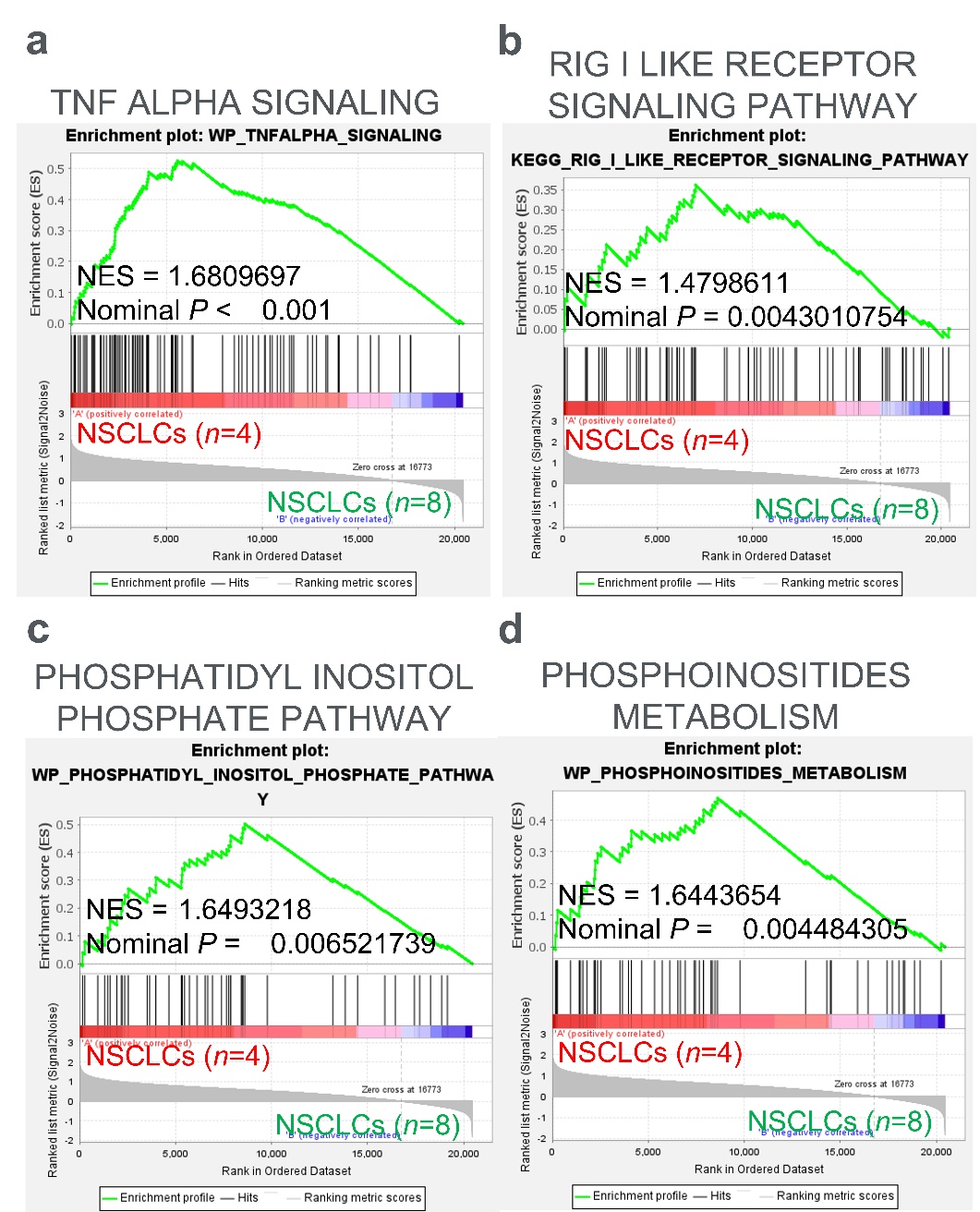
**

**Supplementary Figure 6. GSEA between the 4 patients with up-regulated TLR4, TLR2, TLR1, and PYCR1, and the 8 patients with down-regulated TLR4, TLR2, TLR1, and PYCR1.**

**a**-**d** GSEA was conducted between the 4 patients with up-regulated TLR4, TLR2, TLR1, and PYCR1 (TLR4^up^TLR2^up^TLR1^up^PYCR1^up^ NSCLCs, red) and the 8 patients with down-regulated TLR4, TLR2, TLR1, and PYCR1 (TLR4^down^TLR2^down^TLR1^down^PYCR1^down^ NSCLCs, green). Gene sets related to innate signaling and EGFR signaling are represented (**a**, TNF ALPHA SIGNALING; **b**, RIG I LIKE RECEPTOR SIGNALING PATHWAY; **c**, PHOSPHATIDYL INOSITOL PHOSPHATE PATHWAY; **d**, PHOSPHOINOSITIDES METABOLISM). NES and nominal *P*-value are indicated in the inner panel.


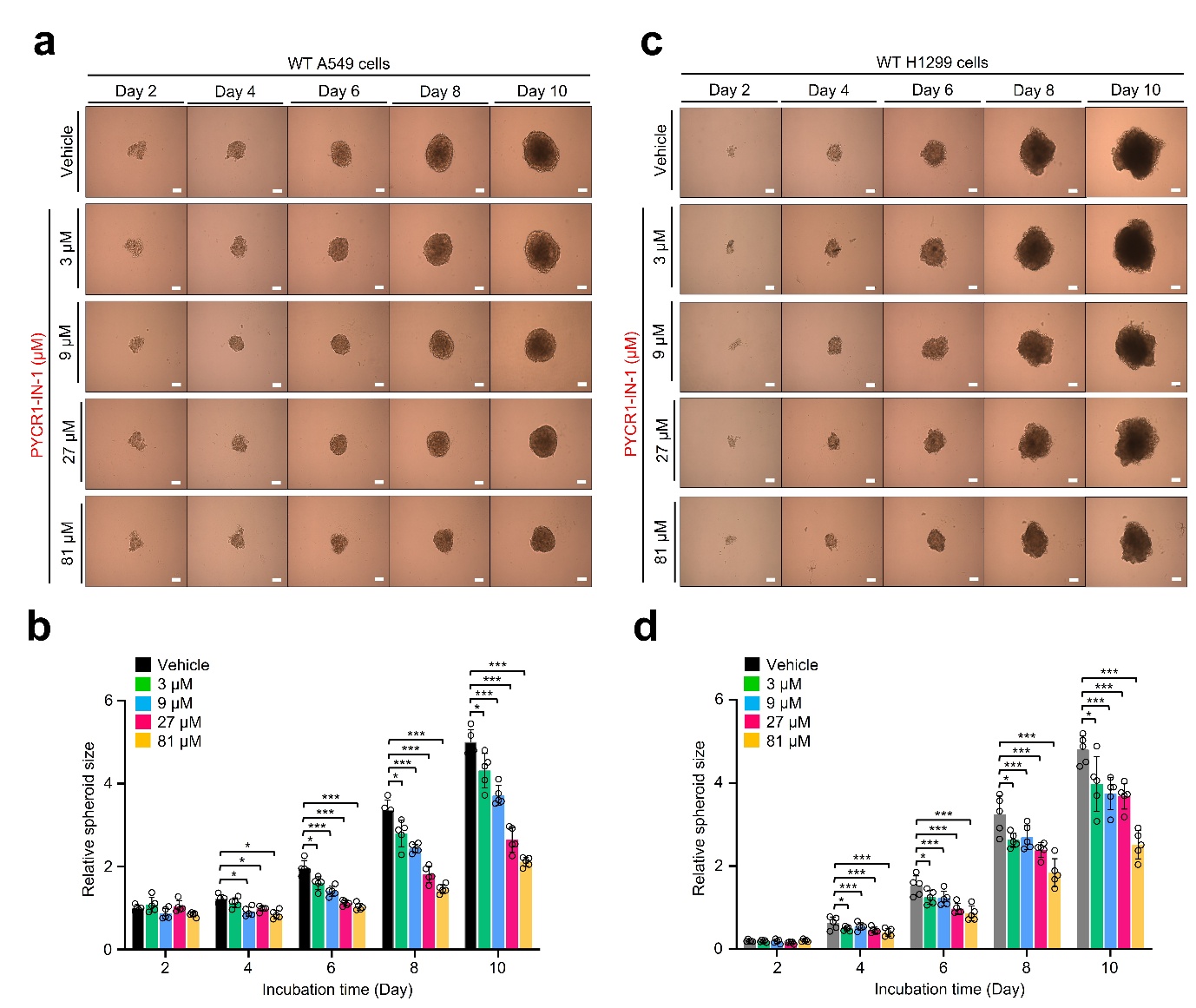


**Supplementary Figure 7.** **PYCR1-IN-1 Inhibits 3D tumor spheroid growth in lung cancer cells.**

**a**-**d** Wild-Type (WT) A549 (250 cells per well, **a** and **b**) or WT H1299 (50 cells per well, **c** and **d**) were seeded into 96-well plates and incubated at 37 °C for 48 hours to allow the formation of 3D spheroids in culture. The spheroids were treated with either vehicle (DMSO, 0.1 % v/v concentration) or different concentrations of PYCR1-IN-1, as indicated. Spheroid sizes were measured using ImageJ Software. Error bars represent ± SD (*n =* 5; **b**, A549; **d**, H1299; scale bar, 100 μm). *, *P* < 0.05; ***, *P* < 0.001.


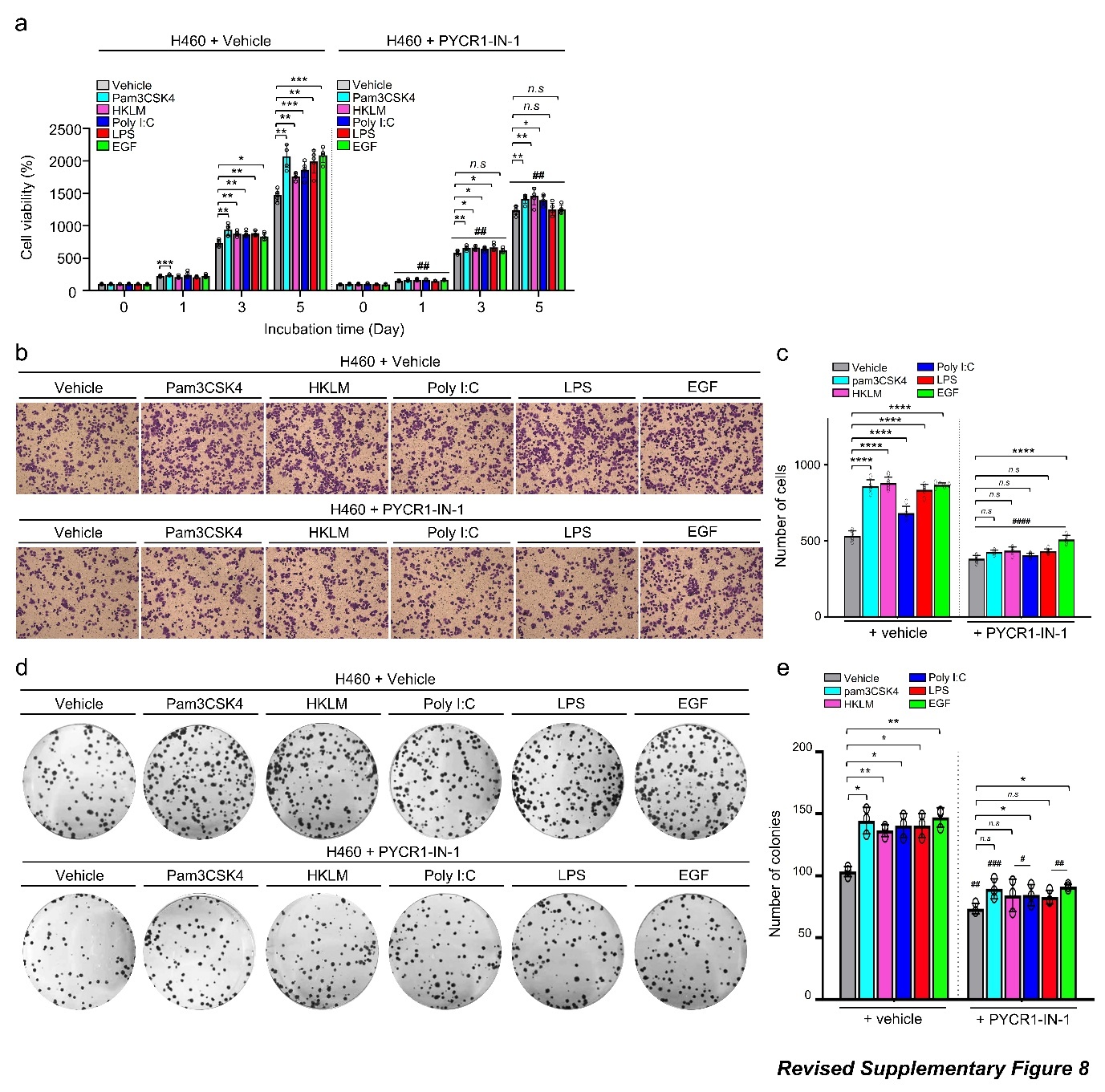


**Supplementary Figure 8.** **PYCR1-IN-1 Inhibits cell proliferation, migration, and anchorage-dependent colony formation of H460 lung cancer cells in response to TLR agonists or EGF.**

**a-e** MTT assay (**a**), transwell migration (**b, c**), and anchorage-dependent colony formation assay (**d, e**) was performed in H460 cells treated with either vehicle (0.1% DMSO) or PYCR1-IN-1 (10 µM) inhibitor, followed by stimulation with vehicle (0.1% DMSO), various TLR agonists - Pam3CSK4 (3 µg/ml), HKLM (10^7^/ml), Poly I:C (5 µg/ml), LPS (5 µg/ml) - and EGF (10 ng/ml). Results are presented as mean ± SD (**a**, *n* = 5; **b, c,** *n* =5; **d, e,** *n* =3). *, *P* < 0.05; **, *P* < 0.01; ***, *P* < 0.001; ****, *P* < 0.0001; ^#^, *P* < 0.05; ^##^, *P* < 0.01; ^###^, *P* < 0.001; ^####^, *P* < 0.0001: H460 treated without PYCR1-IN-1 vs. H460 treated with PYCR1-IN-1. *n.s*, non-significant.


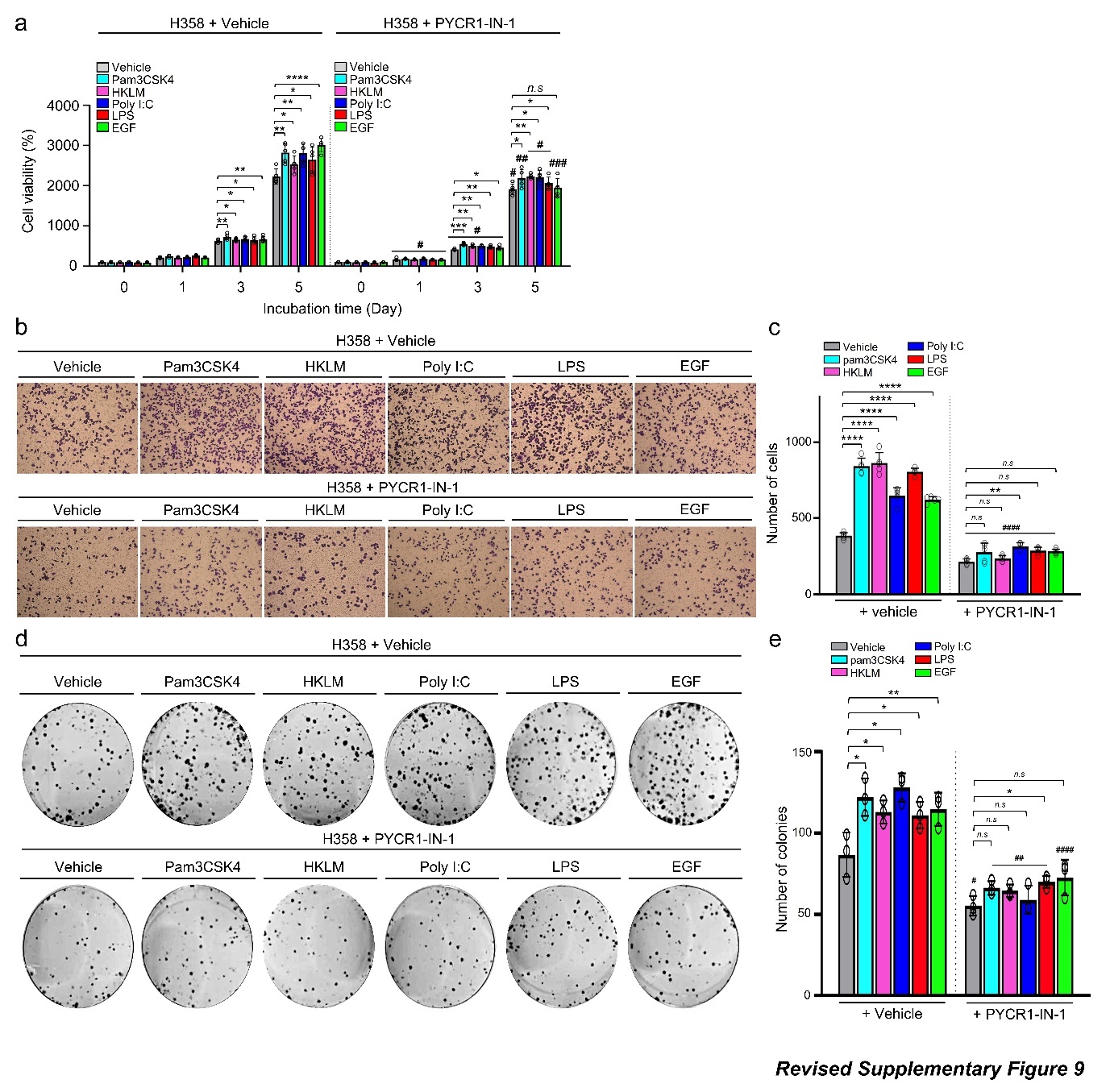


**Supplementary Figure 9.** **PYCR1-IN-1 Inhibits cell proliferation, migration, and anchorage-dependent colony formation of H358 lung cancer cells in response to TLR agonists or EGF.**

**a-e** MTT assay (**a**), transwell migration (**b, c**), and anchorage-dependent colony formation assay (**d, e**) was performed in H358 cells treated with either vehicle (0.1% DMSO) or PYCR1-IN-1 (10 µM) inhibitor, followed by stimulation with vehicle (0.1% DMSO), various TLR agonists - Pam3CSK4 (3 µg/ml), HKLM (10^7^/ml), Poly I:C (5 µg/ml), LPS (5 µg/ml) - and EGF (10 ng/ml). Results are presented as mean ± SD (**a**, *n* = 5; **b, c,** *n* = 5; **d, e,** *n* =3). *, *P* < 0.05; **, *P* < 0.01; ***, *P* < 0.001; ****, *P* < 0.0001; ^#^, *P* < 0.05; ^##^, *P* < 0.01; ^###^, *P* < 0.001; ^####^, *P* < 0.0001: H358 treated without PYCR1-IN-1 vs. H358 treated with PYCR1-IN-1. *n.s*, non-significant.


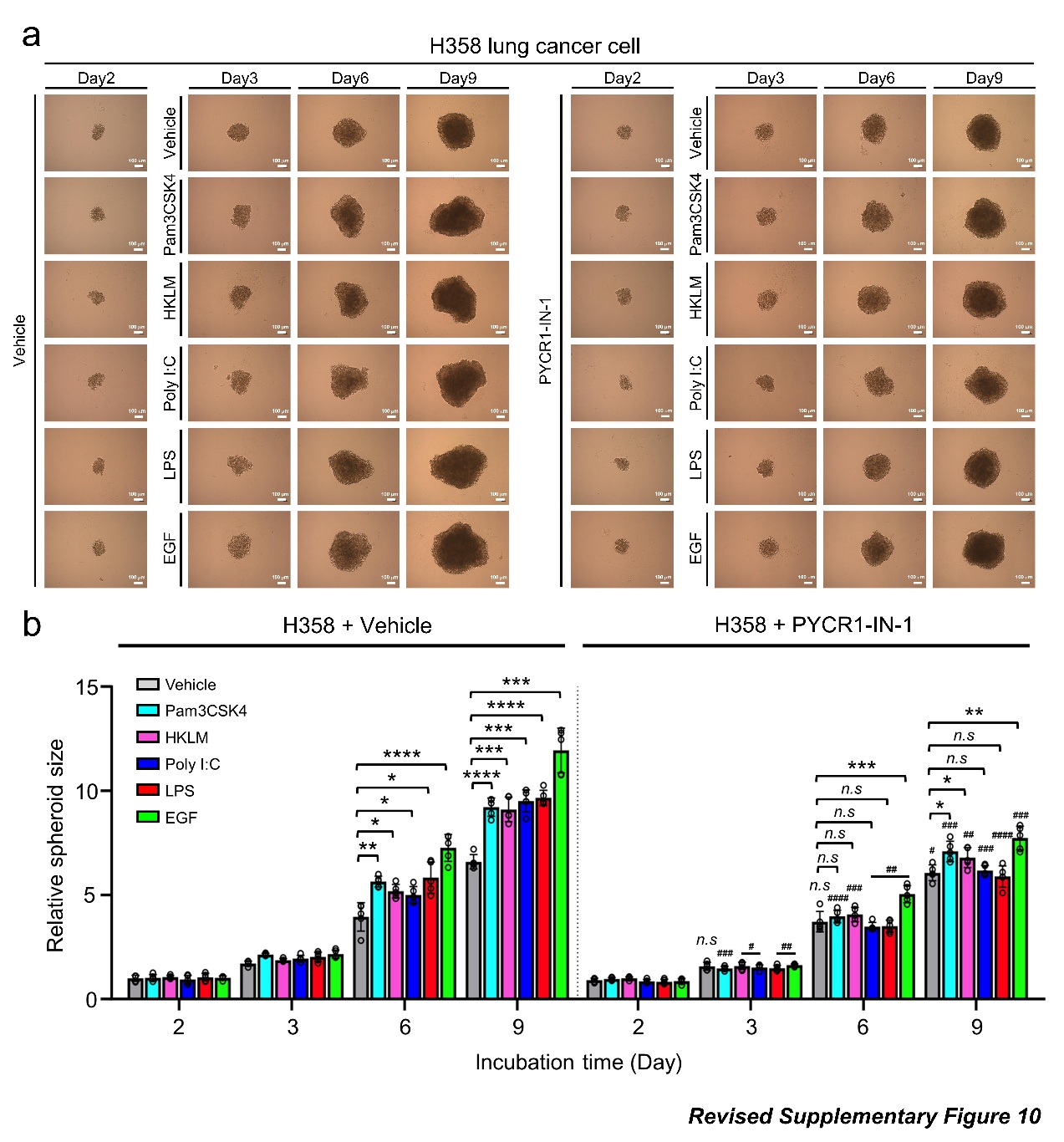


**Supplementary Figure 10.** **PYCR1-IN-1 Inhibits 3D tumor spheroid growth in H358 lung cancer cells.**

**a, b** After stabilization of tumor spheroids derived from H358 cells for 2 days, spheroids were treated with either vehicle (0.1% DMSO) or 10 μM PYCR1-IN-1. After 24 hours, spheroids were further treated with vehicle (0.1% DMSO), Pam3CSK4 (3 µg/mL), HKLM (10^7^/mL), Poly I:C (5 µg/mL), LPS (5 µg/mL), or EGF (10 ng/mL), as indicated. H358-derived tumor spheroid formation and growth were evaluated using phase-contrast microscopy (scale bar, 100 μm) (**a**). Spheroid sizes were measured using ImageJ Software. Error bars represent ± SD (*n =* 5) (**b**). *, *P* < 0.05; **, *P* < 0.01; ***, *P* < 0.001; ****, *P* < 0.0001: ^#^, *P* < 0.05; ^##^, *P* < 0.01; ^###^, *P* < 0.001; ^####^, *P* < 0.0001; H358 spheroids treated without PYCR1-IN-1 vs. H358 spheroids treated with PYCR1-IN-1. *n.s*, non-significant.


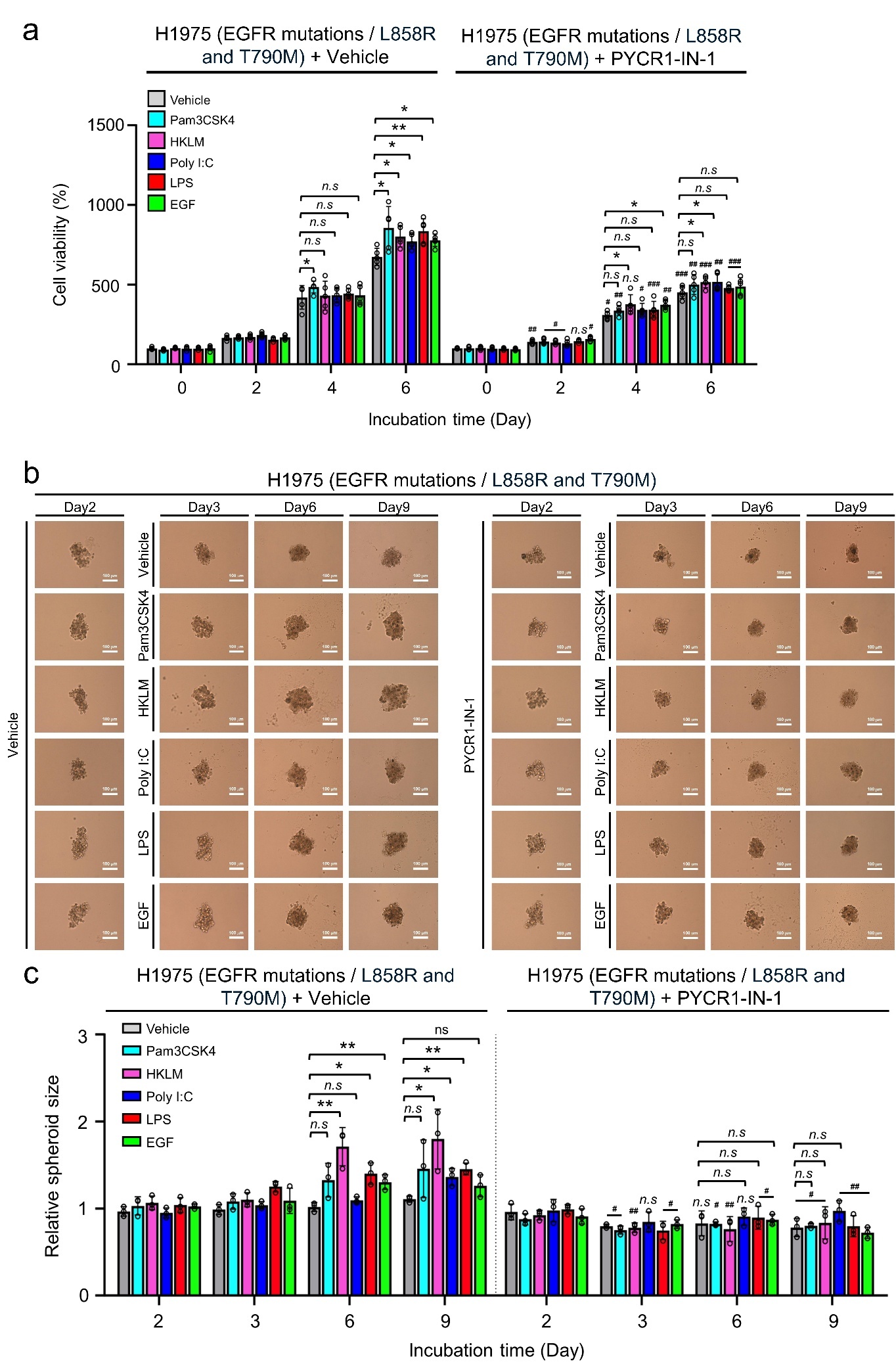


**Supplementary Figure 11.** **PYCR1-IN-1 Inhibits cell proliferation and 3D tumor spheroid growth in** **EGFR mutant** **H1975 (L858R and T790M) lung cancer cells.**

**a** MTT assay was performed in H1975 (EGFR mutations / L858R and T790M) cells treated with vehicle (0.1% DMSO) or PYCR1-IN-1 (10 µM) inhibitor, followed by stimulation with various TLR agonists - Pam3CSK4 (3 µg/ml), HKLM (10^7^/ml), Poly I:C (5 µg/ml), LPS (5 µg/ml) - and EGF (10 ng/ml) for the indicated time periods. Results are presented as mean ± SD (*n* = 5). **b, c** After stabilization of tumor spheroids derived from H1975 (EGFR mutations / L858R and T790M) cells for 2 days, spheroids were treated with 10 μM PYCR1-IN-1. After 24 hours, spheroids were further treated with Pam3CSK4 (3 µg/mL), HKLM (10^7^/mL), Poly I:C (5 µg/mL), LPS (5 µg/mL), or EGF (10 ng/mL), as indicated. H1975-derived tumor spheroid formation and growth were evaluated using phase-contrast microscopy (scale bar, 100 μm) (**a**). Spheroid sizes were measured using ImageJ Software. Error bars represent ± SD (*n =* 3) (**b**). *, *P* < 0.05; **, *P* < 0.01: ^#^, *P* < 0.05; ^##^, *P* < 0.01; ^###^, *P* < 0.001; H1975 treated without PYCR1-IN-1 vs. H1975 treated with PYCR1-IN-1. *n.s*, non-significant.


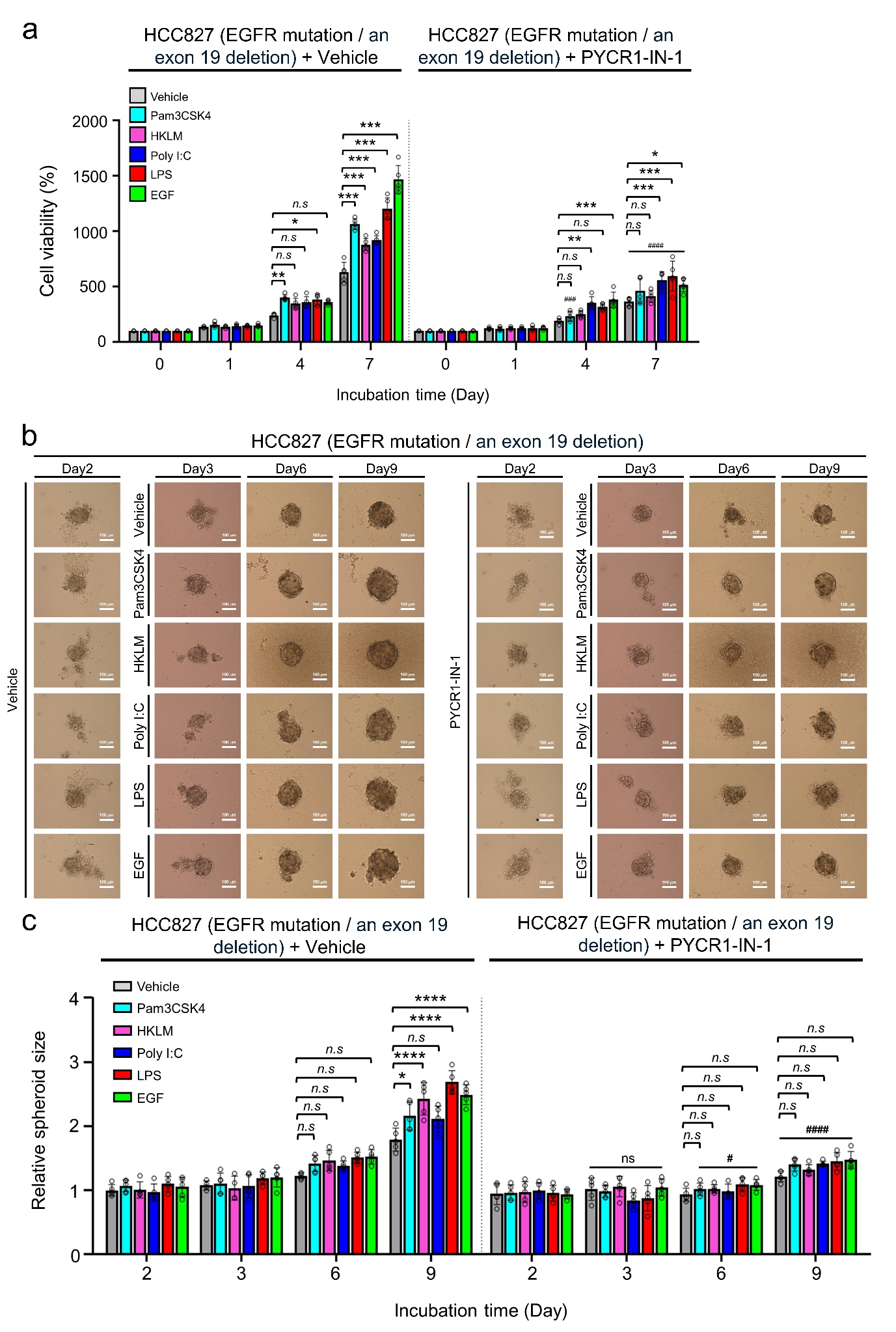


**Supplementary Figure 12.** **PYCR1-IN-1 Inhibits cell proliferation and 3D tumor spheroid growth in** **EGFR mutant HCC827 (an exon 19 deletion) lung cancer cells.**

**a** MTT assay was performed in HCC827 (EGFR mutation / an exon 19 deletion) cells treated with vehicle (0.1% DMSO) or PYCR1-IN-1 (10 µM) inhibitor, followed by stimulation with various TLR agonists - Pam3CSK4 (3 µg/ml), HKLM (10^7^/ml), Poly I:C (5 µg/ml), LPS (5 µg/ml) - and EGF (10 ng/ml) for the indicated time periods. Results are presented as mean ± SD (*n* = 5). **b, c** After stabilization of tumor spheroids derived from EGFR mutant HCC827 (an exon 19 deletion) cells for 2 days, spheroids were treated with 10 μM PYCR1-IN-1. After 24 hours, spheroids were further treated with Pam3CSK4 (3 µg/mL), HKLM (10^7^/mL), Poly I:C (5 µg/mL), LPS (5 µg/mL), or EGF (10 ng/mL), as indicated. HCC827-derived tumor spheroid formation and growth were evaluated using phase-contrast microscopy (scale bar, 100 μm) (**b**). Spheroid sizes were measured using ImageJ Software. Error bars represent ± SD (*n =* 5) (**c**). *, *P* < 0.05; **, *P* < 0.01; ***, *P* < 0.001; ****, *P* < 0.0001: ^#^, *P* < 0.05; ^###^, *P* < 0.001; ^####^, *P* < 0.0001; HCC827 treated without PYCR1-IN-1 vs. HCC827 treated with PYCR1-IN-1. *n.s*, non-significant.

| **Supplementary Table 1. Clinical characteristics of NSCLC patients (n = 42), and differential magnitude (ΔMag) of PYCR1 expression** | | | | | | | |
| --- | --- | --- | --- | --- | --- | --- | --- |
| **between lung tumor tissues (LTTs, n = 42) and matched lung normal tissues (mLNTs, n = 42).** | | | | | | | |
| **Patient** | **Gender** | **Age** | **Histology** | **Stage** | **PYCR1 levels (LTTs,** | **PYCR1 levels (mLNTs,** | **∆Mag (PYCR1)** |
| **ID** |  | **(Year)** | **(Cell type)** |  | **Lung Tumor Tissues)** | **matched Lung Normal Tissues)** | **LTTs vs. mLNTs** |
| **LTT03** | Man | 75 | Others | Stage IIA | 148.2639 | 12.69218 | **135.57172** |
| **LTT20** | Man | 69 | Adenocarcinoma | Stage 1A | 99.06814 | -9.55915 | **108.62729** |
| **LTT07** | Man | 64 | Others | Stage IIA | 107.6882 | 9.739085 | **97.949115** |
| **LTT27** | Man | 77 | Adenocarcinoma | Stage 1A | 78.83222 | 11.26058 | **67.57164** |
| **LTT53** | Man | 64 | Others | Stage 1B | 73.70248 | 15.6991 | **58.00338** |
| **LTT01** | Man | 76 | Squamous cell carcinoma | Stage IIB | 65.23545 | 9.655011 | **55.580439** |
| **LTT42** | Woman | 59 | Adenocarcinoma | Stage IIA | 54.84438 | 8.593657 | **46.250723** |
| **LTT48** | Man | 68 | Adenocarcinoma | Stage IIB | 53.84873 | 15.40193 | **38.4468** |
| **LTT34** | Man | 65 | Adenocarcinoma | Stage 1A | 39.76382 | 6.305965 | **33.457855** |
| **LTT19** | Man | 69 | Others | Stage 1A | 55.4791 | 22.80338 | **32.67572** |
| **LTT25** | Man | 59 | Adenocarcinoma | Stage 1B | 54.49453 | 22.52582 | **31.96871** |
| **LTT28** | Woman | 79 | Adenocarcinoma | Stage 1B | 35.21181 | 3.376407 | **31.835403** |
| **LTT50** | Man | 72 | Squamous cell carcinoma | Stage 1B | 42.21258 | 13.1678 | **29.04478** |
| **LTT29** | Woman | 47 | Adenocarcinoma | Stage 1B | 36.54184 | 8.473656 | **28.068184** |
| **LTT51** | Woman | 73 | Adenocarcinoma | Stage 1B | 21.58927 | -5.72851 | **27.31778** |
| **LTT22** | Man | 84 | Adenocarcinoma | Stage 1B | 56.05973 | 32.20206 | **23.85767** |
| **LTT06** | Man | 67 | Squamous cell carcinoma | Stage 1B | 38.52282 | 17.3229 | **21.19992** |
| **LTT30** | Man | 81 | Adenocarcinoma | Stage 1B | 15.64958 | -5.401637 | **21.051217** |
| **LTT17** | Woman | 68 | Adenocarcinoma | Stage 1B | 31.45391 | 14.06756 | **17.38635** |
| **LTT11** | Woman | 53 | Others | Stage IIA | 45.61076 | 29.05502 | **16.55574** |
| **LTT36** | Man | 70 | Adenocarcinoma | Stage 1B | 21.0813 | 4.80718 | **16.27412** |
| **LTT47** | Man | 63 | Others | Stage 1B | 20.75507 | 4.650098 | **16.104972** |
| **LTT49** | Man | 67 | Squamous cell carcinoma | Stage IIB | 26.78942 | 11.90819 | **14.88123** |
| **LTT14** | Man | 70 | Adenocarcinoma | Stage 1A | 44.87144 | 30.45411 | **14.41733** |
| **LTT13** | Woman | 56 | Adenocarcinoma | Stage 1B | 37.25495 | 30.48073 | **6.77422** |
| **LTT52** | Woman | 43 | Adenocarcinoma | Stage 1B | 18.93566 | 13.6448 | **5.29086** |
| **LTT33** | Man | 75 | Adenocarcinoma | Stage 1A | 10.54639 | 7.539818 | **3.006572** |
| **LTT18** | Woman | 72 | Others | Stage 1B | 31.63409 | 29.52621 | **2.10788** |
| **LTT39** | Man | 68 | Adenocarcinoma | Stage 1B | 20.44698 | 18.89376 | **1.55322** |
| **LTT35** | Woman | 50 | Adenocarcinoma | Stage IIA | 14.01655 | 14.71329 | **-0.69674** |
| **LTT04** | Man | 65 | Others | Stage IIB | 17.89567 | 20.14627 | **-2.2506** |
| **LTT26** | Woman | 38 | Adenocarcinoma | Stage IIA | 4.713228 | 8.062332 | **-3.349104** |
| **LTT43** | Man | 70 | Adenocarcinoma | Stage 1B | 9.940701 | 14.23435 | **-4.293649** |
| **LTT32** | Woman | 66 | Adenocarcinoma | Stage IIA | 3.42613 | 9.072397 | **-5.646267** |
| **LTT02** | Man | 77 | Squamous cell carcinoma | Stage IIB | 24.711 | 30.71867 | **-6.00767** |
| **LTT24** | Man | 70 | Adenocarcinoma | Stage IIB | 33.56794 | 41.84832 | **-8.28038** |
| **LTT38** | Man | 72 | Others | Stage IIA | 2.340727 | 17.7442 | **-15.403473** |
| **LTT05** | Man | 67 | Squamous cell carcinoma | Stage IIIA | 12.86778 | 29.38792 | **-16.52014** |
| **LTT21** | Woman | 60 | Adenocarcinoma | Stage 1B | 31.33384 | 51.70304 | **-20.3692** |
| **LTT08** | Man | 59 | Squamous cell carcinoma | Stage IIB | -2.969096 | 18.16941 | **-21.138506** |
| **LTT12** | Woman | 54 | Adenocarcinoma | Stage 1B | 21.26104 | 52.63665 | **-31.37561** |
| **LTT10** | Woman | 65 | Adenocarcinoma | Stage 1B | 27.23561 | 74.49631 | **-47.2607** |

| **Supplementary Table 2. Differential magnitude (ΔMag) of PYCR1 and EGFR expression between lung tumor tissues (LTTs, n = 42) and matched lung normal tissues (mLNTs, n = 42).** | | | | | | | |
| --- | --- | --- | --- | --- | --- | --- | --- |
|  | | | | | | | |
| **Patient** | **PYCR1 levels (LTTs,** | **EGFR levels (LTTs,** | **PYCR1 levels (mLNTs,** | **EGFR levels (mLNTs,** | **∆Mag (PYCR1)** | **∆Mag (EGFR)** |  |
| **ID** | **Lung Tumor Tissues)** | **Lung Tumor Tissues)** | **matched Lung Normal Tissues)** | **matched Lung Normal Tissues)** | **LTTs vs. mLNTs** | **LTTs vs. mLNTs** |  |
| LTT03 | 148.2639 | 28.55121 | 12.69218 | 104.0612 | 135.57172 | -75.50999 |  |
| **LTT20** | **99.06814** | **3909.546** | **-9.55915** | **90.5948** | **108.62729** | **3818.9512** |  |
| **LTT07** | **107.6882** | **750.5587** | **9.739085** | **137.672** | **97.949115** | **612.8867** |  |
| LTT27 | 78.83222 | 1.652001 | 11.26058 | 166.3085 | 67.57164 | -164.656499 |  |
| **LTT53** | **73.70248** | **288.3939** | **15.6991** | **60.18604** | **58.00338** | **228.20786** |  |
| **LTT01** | **65.23545** | **622.9434** | **9.655011** | **83.47383** | **55.580439** | **539.46957** |  |
| **LTT42** | **54.84438** | **353.4518** | **8.593657** | **138.5836** | **46.250723** | **214.8682** |  |
| **LTT48** | **53.84873** | **131.8352** | **15.40193** | **58.45994** | **38.4468** | **73.37526** |  |
| LTT34 | 39.76382 | 56.79424 | 6.305965 | 139.5723 | 33.457855 | -82.77806 |  |
| **LTT19** | **55.4791** | **556.1363** | **22.80338** | **318.2578** | **32.67572** | **237.8785** |  |
| **LTT25** | **54.49453** | **227.9141** | **22.52582** | **117.7118** | **31.96871** | **110.2023** |  |
| **LTT28** | **35.21181** | **442.8547** | **3.376407** | **119.3026** | **31.835403** | **323.5521** |  |
| LTT50 | 42.21258 | 104.7635 | 13.1678 | 207.9143 | 29.04478 | -103.1508 |  |
| LTT29 | 36.54184 | 127.0354 | 8.473656 | 213.203 | 28.068184 | -86.1676 |  |
| **LTT51** | **21.58927** | **274.3152** | **-5.72851** | **136.4959** | **27.31778** | **137.8193** |  |
| **LTT22** | **56.05973** | **1051.977** | **32.20206** | **280.1729** | **23.85767** | **771.8041** |  |
| **LTT06** | **38.52282** | **607.5068** | **17.3229** | **50.65199** | **21.19992** | **556.85481** |  |
| **LTT30** | **15.64958** | **156.2088** | **-5.401637** | **56.90603** | **21.051217** | **99.30277** |  |
| **LTT17** | **31.45391** | **115.7097** | **14.06756** | **78.3389** | **17.38635** | **37.3708** |  |
| **LTT11** | **45.61076** | **2146.417** | **29.05502** | **783.468** | **16.55574** | **1362.949** |  |
| LTT36 | 21.0813 | 47.87081 | 4.80718 | 172.5178 | 16.27412 | -124.64699 |  |
| **LTT47** | **20.75507** | **216.835** | **4.650098** | **121.8809** | **16.104972** | **94.9541** |  |
| **LTT49** | **26.78942** | **499.5215** | **11.90819** | **97.8767** | **14.88123** | **401.6448** |  |
| **LTT14** | **44.87144** | **305.3798** | **30.45411** | **110.1312** | **14.41733** | **195.2486** |  |
| LTT13 | 37.25495 | 270.6923 | 30.48073 | 383.8595 | 6.77422 | -113.1672 |  |
| **LTT52** | **18.93566** | **206.1025** | **13.6448** | **138.6136** | **5.29086** | **67.4889** |  |
| LTT33 | 10.54639 | 38.55325 | 7.539818 | 103.646 | 3.006572 | -65.09275 |  |
| LTT18 | 31.63409 | 65.97086 | 29.52621 | 224.2608 | 2.10788 | -158.28994 |  |
| **LTT39** | **20.44698** | **197.3099** | **18.89376** | **16.02048** | **1.55322** | **181.28942** |  |
| **LTT35** | **14.01655** | **57.40425** | **14.71329** | **151.7421** | **-0.69674** | **-94.33785** |  |
| LTT04 | 17.89567 | 190.94 | 20.14627 | 154.0914 | -2.2506 | 36.8486 |  |
| LTT26 | 4.713228 | 357.4253 | 8.062332 | 128.8059 | -3.349104 | 228.6194 |  |
| LTT43 | 9.940701 | 121.8054 | 14.23435 | 113.8835 | -4.293649 | 7.9219 |  |
| LTT32 | 3.42613 | 223.3314 | 9.072397 | 78.77055 | -5.646267 | 144.56085 |  |
| LTT02 | 24.711 | 910.1615 | 30.71867 | 112.0902 | -6.00767 | 798.0713 |  |
| LTT24 | 33.56794 | 2799.701 | 41.84832 | 303.1252 | -8.28038 | 2496.5758 |  |
| LTT38 | 2.340727 | 433.8187 | 17.7442 | 149.662 | -15.403473 | 284.1567 |  |
| LTT05 | 12.86778 | 762.3765 | 29.38792 | 170.8964 | -16.52014 | 591.4801 |  |
| LTT21 | 31.33384 | 545.4366 | 51.70304 | 511.0963 | -20.3692 | 34.3403 |  |
| LTT08 | -2.969096 | 153.5461 | 18.16941 | 64.04041 | -21.138506 | 89.50569 |  |
| **LTT12** | **21.26104** | **172.2337** | **52.63665** | **455.7323** | **-31.37561** | **-283.4986** |  |
| **LTT10** | **27.23561** | **131.6197** | **74.49631** | **825.3997** | **-47.2607** | **-693.78** |  |

| **Supplementary Table 3. Differential magnitude (ΔMag) of TRAF6, TAK1, TAB2, and PYCR1 expression between lung tumor tissues (LTTs, n = 42) and matched lung normal tissues (mLNTs, n = 42).** | | | | | | | | | | | | | |
| --- | --- | --- | --- | --- | --- | --- | --- | --- | --- | --- | --- | --- | --- |
| **Patient** | **TRAF6 levels (LTTs,** | **TAK1 levels (LTTs,** | **TAB2 levels (LTTs,** | **PYCR1 levels (LTTs,** | **TRAF6 levels (mLNTs,** | **TAK1 levels (mLNTs,** | **TAB2 levels (mLNTs,** | **PYCR1 levels (mLNTs,** | **∆Mag (TRAF6)** | **∆Mag (TAK1)** | **∆Mag (TAB2)** | **∆Mag (PYCR1)** |  |
| **ID** | **Lung Tumor Tissues)** | **Lung Tumor Tissues)** | **Lung Tumor Tissues)** | **Lung Tumor Tissues)** | **matched Lung Normal Tissues)** | **matched Lung Normal Tissues)** | **matched Lung Normal Tissues)** | **matched Lung Normal Tissues)** | **LTTs vs. mLNTs** | **LTTs vs. mLNTs** | **LTTs vs. mLNTs** | **LTTs vs. mLNTs** |  |
| **LTT03** | **212.3534** | **248.5923** | **127.0615** | **148.2639** | **54.73612** | **8.594965** | **50.32857** | **12.69218** | **157.61728** | **239.997335** | **76.73293** | **135.57172** |  |
| **LTT20** | **244.9199** | **42.76383** | **92.79221** | **99.06814** | **40.22493** | **16.71961** | **32.35375** | **-9.55915** | **204.69497** | **26.04422** | **60.43846** | **108.62729** |  |
| **LTT07** | **210.3134** | **111.496** | **160.0545** | **107.6882** | **71.90968** | **-2.564427** | **49.17806** | **9.739085** | **138.40372** | **114.060427** | **110.87644** | **97.949115** |  |
| **LTT27** | **88.38978** | **69.69079** | **100.7443** | **78.83222** | **81.14362** | **21.75825** | **60.40944** | **11.26058** | **7.24616** | **47.93254** | **40.33486** | **67.57164** |  |
| LTT53 | 42.93336 | 20.28927 | 40.74897 | 73.70248 | 44.59423 | 15.10754 | 11.9416 | 15.6991 | -1.66087 | 5.18173 | 28.80737 | 58.00338 |  |
| **LTT01** | **72.22123** | **19.33587** | **64.06757** | **65.23545** | **48.16367** | **-5.5107** | **26.75299** | **9.655011** | **24.05756** | **24.84657** | **37.31458** | **55.580439** |  |
| LTT42 | 71.52343 | 6.372146 | 36.50737 | 54.84438 | 42.25808 | 10.0834 | 28.93198 | 8.593657 | 29.26535 | -3.711254 | 7.57539 | 46.250723 |  |
| LTT48 | 24.72866 | 4.721373 | 15.75121 | 53.84873 | 39.77121 | 26.02429 | 19.17533 | 15.40193 | -15.04255 | -21.302917 | -3.42412 | 38.4468 |  |
| LTT34 | 43.07645 | 5.509398 | 20.3052 | 39.76382 | 52.28094 | 29.47339 | 32.88225 | 6.305965 | -9.20449 | -23.963992 | -12.57705 | 33.457855 |  |
| LTT19 | 115.886 | 327.6706 | 135.3828 | 55.4791 | 115.9959 | 38.69646 | 67.50009 | 22.80338 | -0.1099 | 288.97414 | 67.88271 | 32.67572 |  |
| LTT25 | 48.05321 | 16.81489 | 67.90546 | 54.49453 | 50.9493 | 20.95493 | 43.23201 | 22.52582 | -2.89609 | -4.14004 | 24.67345 | 31.96871 |  |
| LTT28 | 62.86897 | -4.974117 | 26.79459 | 35.21181 | 54.27541 | 22.6986 | 42.51647 | 3.376407 | 8.59356 | -27.672717 | -15.72188 | 31.835403 |  |
| LTT50 | 73.66717 | 103.3093 | 33.77935 | 42.21258 | 75.14874 | 53.23586 | 30.9785 | 13.1678 | -1.48157 | 50.07344 | 2.80085 | 29.04478 |  |
| LTT29 | 58.30251 | 9.907907 | 35.18677 | 36.54184 | 72.81084 | 35.83873 | 30.13933 | 8.473656 | -14.50833 | -25.930823 | 5.04744 | 28.068184 |  |
| LTT51 | 39.02944 | 6.132792 | 16.24739 | 21.58927 | 53.85136 | 33.93487 | 28.62182 | -5.72851 | -14.82192 | -27.802078 | -12.37443 | 27.31778 |  |
| LTT22 | 109.8141 | 7.824912 | 60.60932 | 56.05973 | 109.1607 | 71.99632 | 66.78143 | 32.20206 | 0.6534 | -64.171408 | -6.17211 | 23.85767 |  |
| **LTT06** | **95.18688** | **72.11982** | **53.23462** | **38.52282** | **32.05526** | **-3.74227** | **33.1509** | **17.3229** | **63.13162** | **75.86209** | **20.08372** | **21.19992** |  |
| **LTT30** | **64.5761** | **20.26623** | **30.09596** | **15.64958** | **57.43921** | **4.251504** | **12.49817** | **-5.401637** | **7.13689** | **16.014726** | **17.59779** | **21.051217** |  |
| LTT17 | 25.10268 | -1.473048 | 27.3994 | 31.45391 | 39.99464 | -1.436831 | 19.30945 | 14.06756 | -14.89196 | -0.036217 | 8.08995 | 17.38635 |  |
| **LTT11** | **95.13357** | **42.71457** | **51.68107** | **45.61076** | **88.35474** | **26.20442** | **42.05257** | **29.05502** | **6.77883** | **16.51015** | **9.6285** | **16.55574** |  |
| LTT36 | 25.46019 | -4.200865 | 30.08623 | 21.0813 | 78.46971 | 24.55686 | 26.81565 | 4.80718 | -53.00952 | -28.757725 | 3.27058 | 16.27412 |  |
| LTT47 | 76.86572 | 33.2834 | 8.802094 | 20.75507 | 44.01225 | 30.87405 | 15.97925 | 4.650098 | 32.85347 | 2.40935 | -7.177156 | 16.104972 |  |
| LTT49 | 76.69601 | 24.88215 | 27.73471 | 26.78942 | 61.39744 | 32.42643 | 10.97563 | 11.90819 | 15.29857 | -7.54428 | 16.75908 | 14.88123 |  |
| LTT14 | 95.16534 | 72.04214 | 163.4111 | 44.87144 | 62.32681 | 9.43151 | 39.56464 | 30.45411 | 32.83853 | 62.61063 | 123.84646 | 14.41733 |  |
| LTT13 | 87.84742 | 54.57286 | 110.6153 | 37.25495 | 130.9618 | 74.11011 | 95.37564 | 30.48073 | -43.11438 | -19.53725 | 15.23966 | 6.77422 |  |
| LTT52 | 48.67517 | 16.64068 | 38.03706 | 18.93566 | 60.72237 | 33.71557 | 35.65038 | 13.6448 | -12.0472 | -17.07489 | 2.38668 | 5.29086 |  |
| LTT33 | 33.88364 | 3.958642 | 25.06948 | 10.54639 | 49.85873 | 14.70962 | 34.72793 | 7.539818 | -15.97509 | -10.750978 | -9.65845 | 3.006572 |  |
| LTT18 | 192.5475 | 68.81826 | 94.32293 | 31.63409 | 124.2108 | 44.63429 | 72.24108 | 29.52621 | 68.3367 | 24.18397 | 22.08185 | 2.10788 |  |
| LTT39 | 18.4473 | -13.55392 | 26.36518 | 20.44698 | 58.29259 | 19.92203 | 30.46658 | 18.89376 | -39.84529 | -33.47595 | -4.1014 | 1.55322 |  |
| **LTT35** | **25.72129** | **-6.654705** | **18.3452** | **14.01655** | **80.63206** | **40.23917** | **25.01932** | **14.71329** | **-54.91077** | **-46.893875** | **-6.67412** | **-0.69674** |  |
| LTT04 | 206.5495 | 39.11879 | 73.02246 | 17.89567 | 89.12452 | 9.681041 | 49.12229 | 20.14627 | 117.42498 | 29.437749 | 23.90017 | -2.2506 |  |
| LTT26 | 65.88937 | 0.2618042 | 9.668291 | 4.713228 | 40.27323 | 12.27525 | 32.86919 | 8.062332 | 25.61614 | -12.0134458 | -23.200899 | -3.349104 |  |
| **LTT43** | **13.01181** | **-2.416031** | **31.17317** | **9.940701** | **57.06775** | **43.63745** | **42.51338** | **14.23435** | **-44.05594** | **-46.053481** | **-11.34021** | **-4.293649** |  |
| LTT32 | 62.11997 | 18.67081 | 17.01057 | 3.42613 | 48.26389 | 24.60163 | 37.3718 | 9.072397 | 13.85608 | -5.93082 | -20.36123 | -5.646267 |  |
| LTT02 | 105.6404 | 36.81621 | 36.5668 | 24.711 | 83.21807 | 36.09723 | 55.05544 | 30.71867 | 22.42233 | 0.71898 | -18.48864 | -6.00767 |  |
| LTT24 | 154.5643 | 28.80106 | 65.2843 | 33.56794 | 58.05913 | 25.32738 | 56.59012 | 41.84832 | 96.50517 | 3.47368 | 8.69418 | -8.28038 |  |
| LTT38 | 90.47238 | 44.04829 | 39.77726 | 2.340727 | 50.9414 | 22.5963 | 17.78899 | 17.7442 | 39.53098 | 21.45199 | 21.98827 | -15.403473 |  |
| LTT05 | 127.7281 | 90.92238 | 86.11266 | 12.86778 | 107.4055 | 18.06905 | 44.59901 | 29.38792 | 20.3226 | 72.85333 | 41.51365 | -16.52014 |  |
| **LTT21** | **57.73475** | **5.939083** | **64.65738** | **31.33384** | **148.1774** | **98.29374** | **169.0193** | **51.70304** | **-90.44265** | **-92.354657** | **-104.36192** | **-20.3692** |  |
| **LTT08** | **15.45009** | **-12.32566** | **26.34424** | **-2.969096** | **40.41865** | **-3.201428** | **59.11334** | **18.16941** | **-24.96856** | **-9.124232** | **-32.7691** | **-21.138506** |  |
| **LTT12** | **29.37901** | **2.230137** | **26.71315** | **21.26104** | **182.5616** | **120.6038** | **148.5523** | **52.63665** | **-153.18259** | **-118.373663** | **-121.83915** | **-31.37561** |  |
| **LTT10** | **109.3095** | **19.1498** | **80.27525** | **27.23561** | **252.824** | **190.2723** | **198.0523** | **74.49631** | **-143.5145** | **-171.1225** | **-117.77705** | **-47.2607** |  |

| **Supplementary Table 4. Differential magnitude (ΔMag) of TLR4, TLR2, TLR1, and PYCR1 expression between lung tumor tissues (LTTs, n = 42) and matched lung normal tissues (mLNTs, n = 42).** | | | | | | | | | | | | |
| --- | --- | --- | --- | --- | --- | --- | --- | --- | --- | --- | --- | --- |
| **Patient** | **TLR4 levels (LTTs,** | **TLR2 levels (LTTs,** | **TLR1 levels (LTTs,** | **PYCR1 levels (LTTs,** | **TLR4 levels (mLNTs,** | **TLR2 levels (mLNTs,** | **TLR1 levels (mLNTs,** | **PYCR1 levels (mLNTs,** | **∆Mag (TLR4)** | **∆Mag (TLR2)** | **∆Mag (TLR1)** | **∆Mag (PYCR1)** |
| **ID** | **Lung Tumor Tissues)** | **Lung Tumor Tissues)** | **Lung Tumor Tissues)** | **Lung Tumor Tissues)** | **matched Lung Normal Tissues)** | **matched Lung Normal Tissues)** | **matched Lung Normal Tissues)** | **matched Lung Normal Tissues)** | **LTTs vs. mLNTs** | **LTTs vs. mLNTs** | **LTTs vs. mLNTs** | **LTTs vs. mLNTs** |
| LTT03 | 116.4391 | -13.12042 | 103.034 | 148.2639 | 118.8646 | -12.90344 | 66.85477 | 12.69218 | -2.4255 | -0.21698 | 36.17923 | 135.57172 |
| **LTT20** | **83.2689** | **24.00914** | **172.6561** | **99.06814** | **25.63334** | **-3.258361** | **4.919621** | **-9.55915** | **57.63556** | **27.267501** | **167.736479** | **108.62729** |
| LTT07 | 75.70138 | -21.28791 | 140.6683 | 107.6882 | 30.03092 | -9.69048 | 12.71665 | 9.739085 | 45.67046 | -11.59743 | 127.95165 | 97.949115 |
| LTT27 | 81.18542 | -13.39182 | 56.49331 | 78.83222 | 115.8488 | 4.757517 | 81.79273 | 11.26058 | -34.66338 | -18.149337 | -25.29942 | 67.57164 |
| LTT53 | 44.8012 | -3.154523 | 28.09278 | 73.70248 | 48.48206 | -3.703789 | 21.7159 | 15.6991 | -3.68086 | 0.549266 | 6.37688 | 58.00338 |
| **LTT01** | **90.58291** | **6.050843** | **155.1146** | **65.23545** | **33.0638** | **-9.694003** | **7.88564** | **9.655011** | **57.51911** | **15.744846** | **147.22896** | **55.580439** |
| LTT42 | 1.090492 | 8.191467 | 15.79302 | 54.84438 | 26.55777 | 2.912867 | 31.48524 | 8.593657 | -25.467278 | 5.2786 | -15.69222 | 46.250723 |
| LTT48 | 11.30269 | -13.24052 | -6.556047 | 53.84873 | 64.8029 | 1.939086 | 23.9832 | 15.40193 | -53.50021 | -15.179606 | -30.539247 | 38.4468 |
| LTT34 | 27.11695 | -15.43007 | -3.394941 | 39.76382 | 89.40562 | 11.39186 | 63.37847 | 6.305965 | -62.28867 | -26.82193 | -66.773411 | 33.457855 |
| LTT19 | 78.16854 | 12.21485 | 168.7466 | 55.4791 | 150.7512 | 21.59582 | 112.6155 | 22.80338 | -72.58266 | -9.38097 | 56.1311 | 32.67572 |
| **LTT25** | **75.29714** | **-2.070966** | **70.60247** | **54.49453** | **50.21067** | **-4.232647** | **34.53753** | **22.52582** | **25.08647** | **2.161681** | **36.06494** | **31.96871** |
| LTT28 | 41.42778 | 14.01068 | 37.93495 | 35.21181 | 100.021 | -0.3241584 | 48.16179 | 3.376407 | -58.59322 | 14.3348384 | -10.22684 | 31.835403 |
| LTT50 | 9.994733 | -5.465766 | 56.92971 | 42.21258 | 97.82095 | 24.10427 | 45.61828 | 13.1678 | -87.826217 | -29.570036 | 11.31143 | 29.04478 |
| LTT29 | -0.0732936 | 4.399683 | -3.249495 | 36.54184 | 55.61167 | 2.548981 | 22.49142 | 8.473656 | -55.6849636 | 1.850702 | -25.740915 | 28.068184 |
| LTT51 | 2.725402 | 55.19246 | 12.75102 | 21.58927 | 60.35207 | 6.901487 | 10.97263 | -5.72851 | -57.626668 | 48.290973 | 1.77839 | 27.31778 |
| LTT22 | 31.66134 | 15.86934 | 14.4647 | 56.05973 | 187.2014 | 4.758834 | 100.6677 | 32.20206 | -155.54006 | 11.110506 | -86.203 | 23.85767 |
| LTT06 | 22.25689 | -10.56626 | 37.15239 | 38.52282 | 42.98742 | -10.85317 | 11.34772 | 17.3229 | -20.73053 | 0.28691 | 25.80467 | 21.19992 |
| LTT30 | 56.54182 | -3.83209 | 18.127 | 15.64958 | 93.29726 | 4.435716 | 27.4686 | -5.401637 | -36.75544 | -8.267806 | -9.3416 | 21.051217 |
| LTT17 | 22.72072 | -1.483565 | 7.06059 | 31.45391 | 47.64342 | -9.366092 | 3.612508 | 14.06756 | -24.9227 | 7.882527 | 3.448082 | 17.38635 |
| LTT11 | 5.051239 | -22.10162 | -3.831451 | 45.61076 | 71.8132 | -5.421449 | 20.40995 | 29.05502 | -66.761961 | -16.680171 | -24.241401 | 16.55574 |
| LTT36 | 7.798307 | -7.808566 | -11.98875 | 21.0813 | 131.1747 | -0.1990874 | 29.41971 | 4.80718 | -123.376393 | -7.6094786 | -41.40846 | 16.27412 |
| LTT47 | 6.811279 | -6.261044 | 6.109809 | 20.75507 | 88.91879 | -4.243575 | 26.71597 | 4.650098 | -82.107511 | -2.017469 | -20.606161 | 16.104972 |
| LTT49 | 31.42113 | -11.9747 | -9.87708 | 26.78942 | 128.1317 | 8.748204 | 37.57532 | 11.90819 | -96.71057 | -20.722904 | -47.4524 | 14.88123 |
| **LTT14** | **184.3722** | **1.024604** | **195.1858** | **44.87144** | **32.20562** | **-5.18411** | **23.02563** | **30.45411** | **152.16658** | **6.208714** | **172.16017** | **14.41733** |
| LTT13 | 64.50797 | 74.05672 | 213.8829 | 37.25495 | 120.5104 | 25.73811 | 148.8543 | 30.48073 | -56.00243 | 48.31861 | 65.0286 | 6.77422 |
| LTT52 | 20.3118 | 8.060923 | 30.23852 | 18.93566 | 39.68243 | 2.149506 | 11.40931 | 13.6448 | -19.37063 | 5.911417 | 18.82921 | 5.29086 |
| LTT33 | 12.03174 | -9.613334 | 16.17071 | 10.54639 | 38.81673 | -10.17545 | 7.92125 | 7.539818 | -26.78499 | 0.562116 | 8.24946 | 3.006572 |
| LTT18 | 65.33083 | -3.294769 | 33.20116 | 31.63409 | 147.3634 | 9.086266 | 120.4458 | 29.52621 | -82.03257 | -12.381035 | -87.24464 | 2.10788 |
| LTT39 | -12.03278 | -8.939546 | -0.5026448 | 20.44698 | 55.79289 | -6.338864 | 32.34225 | 18.89376 | -67.82567 | -2.600682 | -32.8448948 | 1.55322 |
| LTT35 | 49.97831 | -14.32126 | 6.937764 | 14.01655 | 93.39867 | 0.04427625 | 1.602523 | 14.71329 | -43.42036 | -14.36553625 | 5.335241 | -0.69674 |
| LTT04 | 99.41924 | -22.25351 | 21.28594 | 17.89567 | 100.5673 | 0.9347105 | 13.52841 | 20.14627 | -1.14806 | -23.1882205 | 7.75753 | -2.2506 |
| **LTT26** | **-5.623194** | **-11.78165** | **-15.32253** | **4.713228** | **29.04507** | **16.03239** | **25.18264** | **8.062332** | **-34.668264** | **-27.81404** | **-40.50517** | **-3.349104** |
| **LTT43** | **11.18273** | **-13.78003** | **2.838291** | **9.940701** | **128.2398** | **7.111102** | **111.7648** | **14.23435** | **-117.05707** | **-20.891132** | **-108.926509** | **-4.293649** |
| **LTT32** | **33.83539** | **-9.123438** | **5.984518** | **3.42613** | **52.3441** | **7.496103** | **19.75176** | **9.072397** | **-18.50871** | **-16.619541** | **-13.767242** | **-5.646267** |
| **LTT02** | **12.57382** | **-14.50276** | **2.140661** | **24.711** | **50.55797** | **-4.137121** | **13.18955** | **30.71867** | **-37.98415** | **-10.365639** | **-11.048889** | **-6.00767** |
| LTT24 | 45.74575 | 24.0082 | 46.0024 | 33.56794 | 105.5657 | 6.157336 | 90.25772 | 41.84832 | -59.81995 | 17.850864 | -44.25532 | -8.28038 |
| LTT38 | 48.24511 | -5.390982 | 39.97049 | 2.340727 | 54.68033 | -7.284706 | 21.62779 | 17.7442 | -6.43522 | 1.893724 | 18.3427 | -15.403473 |
| LTT05 | 113.4038 | -14.83079 | 297.9858 | 12.86778 | 150.6057 | 0.8916531 | 67.90688 | 29.38792 | -37.2019 | -15.7224431 | 230.07892 | -16.52014 |
| **LTT21** | **17.83532** | **8.581036** | **9.246906** | **31.33384** | **273.1962** | **24.0506** | **268.2405** | **51.70304** | **-255.36088** | **-15.469564** | **-258.993594** | **-20.3692** |
| **LTT08** | **-6.68653** | **-23.83442** | **-9.378112** | **-2.969096** | **33.21156** | **4.467453** | **8.868217** | **18.16941** | **-39.89809** | **-28.301873** | **-18.246329** | **-21.138506** |
| **LTT12** | **3.645279** | **-5.555511** | **12.22509** | **21.26104** | **175.569** | **20.59355** | **204.6988** | **52.63665** | **-171.923721** | **-26.149061** | **-192.47371** | **-31.37561** |
| **LTT10** | **127.7456** | **-6.519569** | **102.0124** | **27.23561** | **398.2639** | **65.57564** | **464.6543** | **74.49631** | **-270.5183** | **-72.095209** | **-362.6419** | **-47.2607** |
